# Haplotype-Resolved Genome of the Critically Endangered, Paleo-endemic Tree, *Eidothea hardeniana*

**DOI:** 10.1093/gbe/evag071

**Published:** 2026-03-19

**Authors:** Abhishek Soni, Agnelo Furtado, Maurizio Rossetto, Robert J Henry

**Affiliations:** ARC Centre of Excellence for Plant Success in Nature and Agriculture, The University of Queensland, St Lucia, QLD 4072, Australia; Centre for Crop Science, Queensland Alliance for Agriculture and Food Innovation, The University of Queensland, St Lucia, QLD 4072 Australia; ARC Centre of Excellence for Plant Success in Nature and Agriculture, The University of Queensland, St Lucia, QLD 4072, Australia; Centre for Crop Science, Queensland Alliance for Agriculture and Food Innovation, The University of Queensland, St Lucia, QLD 4072 Australia; Research Centre for Ecosystem Resilience, Royal Botanic Garden Sydney, Sydney, NSW 2000, Australia; Queensland Alliance for Agriculture and Food Innovation, The University of Queensland, St Lucia, QLD 4072 Australia; ARC Centre of Excellence for Plant Success in Nature and Agriculture, The University of Queensland, St Lucia, QLD 4072, Australia; VinUni Big Data Research Institute, VinUniversity, Hanoi, Vietnam

**Keywords:** *Eidothea*, proteaceae, inbreeding, conservation, genome, IUCN

## Abstract

A fully phased, chromosome-level reference genome assembly for an IUCN-declared critically endangered and paleo-endemic tree, *Eidothea hardeniana* (Nightcap Oak) was assembled de novo using long-read PacBio HiFi, Oxford Nanopore (ONT) and Hi-C sequencing technologies. Consensus assembly represented high contiguity (N50 ∼ 49 Mb), (BUSCO 99.2%), assembly size in accordance with flow cytometry derived genome size estimate, and the presence of telomeric and centromeric repeats on pseudochromosomes. Haplotype 1 (598 Mb) achieved an N50 of 45.8 Mb and 99.1% BUSCO completeness, while Haplotype 2 (596 Mb) showed similar quality (N50: 47.3 Mb; BUSCO: 98.4%). Repeat content comprised approximately 54% of the genome. Gene annotation confirmed approximately 30120 protein coding genes with BUSCO completeness of 99.5%. Comparative orthology with other assembled Proteaceae genomes identified ∼13,000 shared ortholog clusters and 227 *Eidothea*-specific orthologs clusters. Heterozygosity in *E. hardeniana* was estimated at 1.17 SNVs per kilobase (0.12%), which is approximately one-third of the level reported for the other sequenced critically endangered Proteaceae species, *Macadamia jansenii*. This genome represents the first assembled genome from the Australian lineage of the Proteoideae subfamily within Proteaceae. As a high-quality reference for conservation genomics, it provides a foundation for SNP genotyping, population structure analyses, and the development of informed genetic rescue strategies for *E. hardeniana* and related species.

Significance This study presents the first chromosome-scale, haplotype-resolved reference genome for *Eidothea hardeniana*, an IUCN-listed critically endangered Australian species from the Proteoideae clade of the Proteaceae family. This genome provides an essential resource for phylogenomics, conservation genetics, genetic diversity assessments, and studies of genome evolution in *E. hardeniana* and the wider Proteaceae.

## Introduction

*Eidothea hardeniana* P.H Weston & Kooyman, commonly Nightcap Oak, an IUCN declared critically endangered plant species, was rediscovered in early 2000s (Weston and Kooyman 2002). The genus *Eidothea* is classified in the Proteoideae subfamily of the Proteaceae, which consists of 27 genera and 100 species found in a wide range of habitats in the southern hemisphere (Rossetto and Kooyman 2005; Weston and Barker 2006; Sauquet et al. 2009).

*Eidothea hardeniana* is considered a paleo-endemic species, once more widespread but now confined to a narrow 8 to 10 km^2^ area in New South Wales, Australia with approximately 250 individuals (Rossetto and Porter 2005). Its historical decline has been attributed to climatic shifts and habitat fragmentation over evolutionary timescales (Rossetto and Kooyman 2005; Rossetto and Porter 2005). The small population exhibits a limited capacity for seed dispersal and recruitment (Rossetto and Kooyman 2005). These characteristics, combined with low census numbers, make *E. hardeniana* especially vulnerable to inbreeding and genetic erosion. Understanding the genetic structure of the populations is critical for effective conservation. The lack of a suitable reference genome has been a major barrier to assessing key parameters such as genomic diversity, demographic bottlenecks, and functional gene retention, factors critical for informing conservation interventions.

Here we report a haplotype-resolved, chromosome-scale reference genome for *E. hardeniana*. The genome was assembled using Pacific Biosciences High Fidelity (HiFi) reads (Fig. S1) and Hi-C data, with extra-long Oxford Nanopore (ONT) sequencing reads (Fig. S2, Nurk et al. 2022; Shang et al. 2023; Espinosa et al. 2024; Lu et al. 2024). The resulting reference genome provides a crucial foundation for investigating genetic load, gene content, and genome architecture in an evolutionarily isolated lineage.

## Results and Discussion

### Genome Size Estimate and Ploidy Analysis

Flow cytometry-based genome size estimate for *E. hardeniana* was 1C = 605 Mbp (Fig. S3). K-mer-based size estimates varied significantly from 550 to 770 Mbp (Figs. S4 and S5). Using short k-mer (21), the ploidy inferred by Smudgeplot was tetraploid (Fig. S6), while using the best predicted k-mer (127) by Kmergenie, the Smudgeplot predicted a diploid (Fig. S7). Importantly, the flow cytometry estimate (∼605 Mbp) closely matched the final assembly size, supporting interpretation of the sampled individual as diploid. This also corrected earlier overestimates (1C = 887 Mbp) that likely resulted from inaccurate reference standards (e.g. *Pisum sativum*) used in previous FCM analyses (Veselý et al. 2011; Šmarda et al. 2014).

### Genome Assembly

The HiFi + Hi-C assembly produced five T2T scaffolds. Incorporating unfiltered ONT reads, this assembly consisted of one T2T contig of chromosome length (Fig. S8, Table S1). While scaffolding achieved six T2T scaffolds. Filtering ONT reads to >20, >30 and >40 kb further reduced fragmentation at contig level assembly as the number of contigs went down from 1684 to 670 (Table S2), likewise the scaffolds number reduction from 1701 to 644 indicated less fragmentation at scaffold-level assembly. Assemblies incorporating ONT reads up to 40 kb yielded five to six chromosome-scale T2T scaffolds (Figs. S9 to S14). Whereas, the integration of extra-long ONT reads (>50 kb) produced 14 chromosome-length scaffolds, 10 of which contained telomeric repeats at both ends (Figs. S9 and S15). Hi-C contact map supported the chromosome-scale scaffolding and identified centromeric interaction patterns (Fig. S16). Four scaffolds contained telomeric repeats at only one end (Fig. S15), and were therefore, considered candidate chromosome arms. Based on (i) strong collinearity to *Protea cynaroides* genome (Fig. S17), and Hi-C interaction support for adjacency and orientation (Fig. S18 to S19), these scaffolds were joined during manual curation into two chromosome-scale pseudochromosomes. Subsequently, telomeric motifs (TTTAGGG) were identified at both ends of all manually joined scaffolds (Fig. S20), supporting their structural completeness. Hi-C contact maps (Figs. S21 and S22) and centromere predictions (Fig. S23) further supported that all 12 pseudochromosomes showed a single major centromeric signal except chromosome 1, which showed two centromere-like peaks (Fig. S22); in the absence of karyological confirmation, we interpret this cautiously as either a dicentric configuration or an artefact of centromere inference or scaffolding. Due to the critically endangered status of *E. hardeniana*, karyotyping was not feasible, necessitating reliance on the genome assembly, Hi-C contact maps, and comparative collinearity to infer chromosome structure.

The 12 pseudochromosomes in the assembly accounted for the 99.2% BUSCO with a total length of 614 Mb and N50 of 49 Mb (Table 1). Smaller unplaced scaffolds (69 Mb) were identified containing organellar DNA (mitochondrial and chloroplast DNA) (Fig. S24 to S26). The final assembly was aligned with *P. cynaroides* and *Macadamia jansenii* (Fig. S27 and S28). As expected, being in the same subfamily, Proteoideae, *E. hardeniana* showed more resemblance to the *P. cynaroides* genome.

**Table 1:** Assembly statistics of the final consensus and haplotype genome assemblies and comparison against other Proteaceae genomes

Quality parameters	*Eidothea hardeniana*			*Macadamia jansenii (Sharma et al. 2024)*	*Telopea speciosissima (Chen et al. 2022)*
	Consensus	Haplotype 1	Haplotype 2		
Number of scaffolds	12	12	12	14	11
Total size (Mb)	614.2	598.1	595.9	773	823
Min length (Mb)	40	39.8	38.4	-	-
Max length (Mb)	79.5	73.3	74.9	-	-
N90 (Mb)	40.4	39.8	39.9	-	-
N80 (Mb)	44.6	43.7	43.9	-	-
N70 (Mb)	45.3	44.4	44.1	-	-
N60 (Mb)	47.8	45.8	47.1	-	-
N50 (Mb)	48.9	45.8	47.3	54.7	69
Average length (Mb)	51.2	48.2	50.1	-	-
Number of contigs	26	35	36	-	-
Contig N50 (Mb)	24	23	23.5	-	-
Percent gaps (%)	0.00%	0.00%	0.00%	-	-
BUSCO					
Complete (C)	99.20%	99.10%	98.40%	97.70%	97.80%
Single copy (S)	86.70%	87.00%	86%	84.20%	86.70%
Duplicated (D)	12.50%	12.10%	12.40%	13.50%	11.20%
Fragmented (F)	0.20%	0.20%	0.20%	0.70%	1.70%
Missing (M)	0.60%	0.70%	1.40%	1.60%	0.50%

Both haplotypes exhibited >98% BUSCO completeness against the embryophyta_odb10 dataset. Dot plot alignments confirmed high collinearity between the two haplotypes and the consensus genome (Figs. S29 to S30), indicating structurally consistent chromosome-scale assemblies.

### Repeat Content Analysis

The consensus genome contained 53.9% repetitive DNA, of which a substantial proportion (∼33%) remained unclassified (Table S3). Both haplotypes similarly comprised just over half of their genomic sequence as repetitive DNA (53.2% in Hap1 and 53.4% in Hap2). The repeat fraction was dominated by retrotransposons, particularly LTR elements (14.38%), with Gypsy/DIRS1 (10.8%) and Ty1/Copia (2.52%) families representing the major components. LINEs accounted for approximately 3.1% of the genome, largely from L1/CIN4 subfamilies (2.28%). Overall, the repeat landscapes of the two haplotypes were highly similar (Table S4 to S5).

### Structural Annotation and Gene Model Quality

Structural annotation identified 30,411 protein-coding sequences in the consensus assembly (Table S6). Comparison of the two haplotype assemblies indicated a high degree of similarity in gene content and genomic architecture, with only minor differences (Table S6). A total of 29,712 and 29,416 coding sequences were predicted in Hap1 and Hap2, respectively. Assessment of gene model completeness using BUSCO against the embryophyta_odb10 database indicated 97.7% completeness for the consensus genome assembly, with 10.3% classified as complete and duplicated BUSCOs (Table S7).

### Functional Annotation of the Genome

Functional annotation of the 30,120 identified protein-coding sequences assigned at least one Gene Ontology (GO) term to 24,649 genes (Table S8). Among the 548 genes without BLAST matches, 449 were supported as protein-coding by coding-potential analysis. Comparison of the two haplotype assemblies (Hap1 and Hap2) revealed a high degree of concordance in gene content and annotation. In Hap1, from 29,712 coding sequences, 24,304 were annotated with at least one GO term (Table S8). Whereas in Hap2, from 29,416 predicted coding sequences, 24,011 received at least one GO annotation.

### Comparative Genomics and Orthologous Gene Analysis

A total of 67,982 orthologs clusters were identified across all species from which 13,086 orthologs clusters were shared across all four species (Fig. S31). Only 227 unique orthologs clusters were identified for the *E. hardeniana* (Fig. S31). These, orthologs may reflect lineage-specific gene retention or divergence since its early separation from the other Proteaceae lineages (Fig. S32). Comparative orthology analysis across Proteaceae revealed 227 species-specific orthogroups in *E. hardeniana*.

### Synteny and Collinearity

**Fig. 1.**
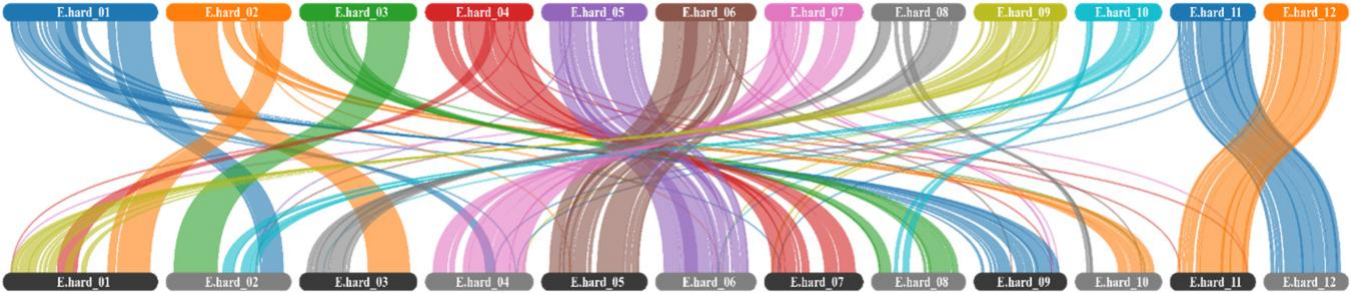
Syntenic relationships within the Eidothea hardeniana genome based on chromosome-level scaffolds of the final consensus assembly. Ribbons connect syntenic gene blocks between chromosomes, revealing patterns of ancient segmental duplication and potential paralogy.

Self-synteny analysis revealed limited evidence of ancient whole genome duplication followed by rearrangements within the genome (Fig. 1). Chromosomes 5, 6, 11 and 12 indicated comparatively lower chromosomal rearrangement than others (Fig. 1) with strong collinearity and structural conservation between haplotypes (Fig. S33).

Structural comparison between the two haplotype-resolved assemblies of *Eidothea hardeniana* using SyRI revealed largely conserved genome architecture with only a limited number of structural rearrangements (Fig. S34). Comparative analyses between *E. hardeniana* and *P. cynaroides* revealed both deeply conserved synteny and substantial rearrangements (Fig. S35 to S36).

### Antimicrobial Peptides Genes

Genes with homology to antimicrobial peptides (AMPs) in *Eidothea hardeniana* exhibited structural conservation despite a high degree of genome divergence. The g686.t1 transcript in *E. hardeniana* contained three copies of the conserved cysteine motif, in contrast to four copies in *Macadamia integrifolia* and *M. jansenii*. The *E. hardeniana* AMP was shorter, consisting of 543 amino acids compared with 636 amino acids in *M. integrifolia* (AF161884.1) (Fig. S37) (Marcus et al. 1999; Sharma et al. 2021, 2024).

### Variant Calling and Heterozygosity

The number of SNVs (single nucleotide variants) was assessed across a range of allele frequency thresholds. As the minimum frequency threshold for variant detection decreased from 40% to 15%, the total number of variant positions increased from 417,292 to 720,563 (Fig. S38). This asymptotic increase indicated saturation of high-confidence variant detection at lower frequency thresholds, supporting the robustness of heterozygosity estimates. Based on the plateau observed below 20% frequency threshold, the heterozygosity rate was estimated to be approximately 1.17 SNVs per kilobase or 0.12%, considerably lower than in related Proteaceae species such as *Protea cynaroides* (1.07%) (Chang et al. 2023), *M. integrifolia* (1.55%) (Sharma et al. 2024), *M. tetraphylla* (1.33%) (Sharma et al. 2024), *M. jansenii* (0.31%) (Sharma et al. 2021), and *Telopea speciosissima* (0.76%) (Chen et al. 2022). After refining for 100% homozygous and high-confidence heterozygous calls, 719,174 SNV positions were retained. The low heterozygosity observed in *E. hardeniana* is likely a consequence of prolonged demographic isolation and a persistently small population size. Despite this, the estimated heterozygosity indicates that the species retains a reservoir of genetic diversity that may be important for future adaptability. However, the restricted population size suggests that inbreeding and genetic drift continue to influence genomic variation. The genetic distinctiveness of each surviving individual further highlights the conservation significance of the species, as the loss of any single tree may result in the irreversible loss of unique alleles from the gene pool.

## Materials and Methods

### Genome Size and Ploidy Estimation

Young green leaves of *Eidothea hardeniana* (accession AA 20010147) were collected from the Royal Botanic Garden Sydney, Australia. Genome size was estimated using both flow cytometry (FCM) and k-mer approaches. For FCM, *E. hardeniana* leaf tissue was co-chopped with Nipponbare rice following established protocols (Soni and Henry 2025). Fluorescence was measured as described previously (Soni et al. 2025; Soni and Henry 2025). For k-mer analyses, Kmergenie was used to select the best k-mer and obtain an initial estimate (Chikhi and Medvedev 2014). Subsequently, GenomeScope 2.0 was used to estimate genome size and heterozygosity (Ranallo-Benavidez et al. 2020). Jellyfish and Smudgeplot were used to evaluate ploidy scenarios (Marçais and Kingsford 2011; Ranallo-Benavidez et al. 2020) (Supplementary Methods S1).

### DNA/RNA Sequencing and QC

High molecular weight DNA was extracted using a modified CTAB method (GIH_SOP204-02, https://dx.doi.org/10.17504/protocols.io.b5qyq5xw), and long-read sequencing (PacBio HiFi, ONT), Hi-C sequencing, and short-read WGS were generated. Read QC used FastQC; adapter/quality trimming used fastp (Chen et al. 2018; Chen 2023), and HiFi reads were screened for adapter contamination. RNA was extracted using modified CTAB-based workflows (Wang and Stegemann 2010; Rubio-Piña and Zapata-Pérez 2011) and QC-filtered with fastp (Chen et al. 2018; Chen 2023).

### Assembly, Curation, Annotation, and Comparative Analyses

Haplotype-resolved assemblies were generated using HiFiasm under multiple ONT-integration strategies (Cheng et al. 2021), and Hi-C scaffolding used YaHS (Zhou et al. 2023) after mapping Hi-C reads with bwa (Li 2013). Scaffolding was validated using dot plots (Cabanettes and Klopp 2018) and curated by joining highly collinear scaffolds. Assembly quality and metrics were assessed using BUSCO and QUAST (Gurevich et al. 2013; Simão et al. 2015; Seppey et al. 2019), and Hi-C contact maps were inspected using standard Hi-C visualization workflows (Durand et al. 2016a, 2016b; Wolff et al. 2022). Repeats were identified with RepeatModeler2 and masked with RepeatMasker (Chen 2004; Flynn et al. 2020). Genes were predicted with BRAKER3 (Gabriel et al. 2024) using RNA-seq evidence and Viridiplantae peptides (OrthoDB_v11) obtained from (https://www.orthodb.org), and were functionally annotated with BLAST/GO/InterPro approaches (Conesa et al. 2005; Jones et al. 2014). Variant calling and heterozygosity was estimated using short reads, mapped under multiple alignment stringencies in CLC Genomics Workbench. Comparative Orthology was conducted using OrthoFinder (Emms and Kelly 2019). Synteny was inferred using DIAMOND and MCScanX (Wang et al. 2012; Buchfink et al. 2021). Chromosomal rearrangements were inferred with minimap2 and SyRI (Li 2018; Goel et al. 2019). Full protocols, software versions, and parameter settings are provided in Supplementary Methods S1.

## Supplementary Material

evag071_Supplementary_Data

## Data Availability

All sequencing data for *Eidothea hardeniana* are deposited at NCBI under BioProject PRJNA1330521 with raw reads linked to BioSample SAMN51526000. Short-read WGS (SRR35515829), RNA-seq (SRR35515830), Hi-C (SRR35515831), PacBio HiFi (SRR35515833), and ONT long reads (SRR35534503) are available at the Sequence Read Archive (SRA). Genome assemblies include the consensus assembly (SUB15652535), haplotype 1 (SUB15653222), and haplotype 2 (SUB15653255). figures are supplied as separate files. For peer review, data are available via NCBI reviewer access: BioProject PRJNA1330521 (reviewer link): https://dataview.ncbi.nlm.nih.gov/object/PRJNA1330521?reviewer=7hq106mjmcog25t5lto0jf7pn9 SRA accessions: WGS (SRR35515829), RNA-seq (SRR35515830), Hi-C (SRR35515831), PacBio HiFi (SRR35515833), ONT (SRR35534503). figures accompany the manuscript.
