# Supplementary material for: Haplotype-Resolved Genome of the Critically Endangered, Paleo-endemic Tree, Eidothea hardeniana
Source: Genome Biol Evol. 2026 Mar 19;18(4):evag071. doi: 10.1093/gbe/evag071 (PMC13080361; doi:10.1093/gbe/evag071)
Supplement: evag071_Supplementary_Data [file evag071_supplementary_data.zip › Supplementary_figures_without_track_changes.docx]

**Supplementary Figures (S1-S38)**

**Haplotype-resolved genome of the critically endangered, paleo-endemic tree, *Eidothea hardeniana***

Authors: Abhishek Soni^1,2^, Agnelo Furtado^1,2^, Maurizio Rossetto^3,4^, Robert J. Henry^1,5*^

[^a.soni@uq.edu.au (A.S.)^](mailto:a.soni@uq.edu.au%20(A.S.))^,^ [^a.furtado@uq.edu.au^](mailto:a.furdato@uq.edu.au) ^(A.F.),^ [^maurizio.rossetto@botanicgardens.nsw.gov.au^](mailto:maurizio.rossetto@botanicgardens.nsw.gov.au) ^(M.R.),^ [^robert.henry@uq.edu.au^](mailto:robert.henry@uq.edu.au) ^(R.J.H.)^

1. ARC Centre of Excellence for Plant Success in Nature and Agriculture, The University of Queensland, St Lucia 4072 QLD Australia
2. Centre for Crop Science, The Queensland Alliance for Agriculture and Food Science, The University of Queensland, St Lucia 4072 QLD Australia
3. Research Centre for Ecosystem Resilience, Royal Botanic Gardens, Sydney 2000 NSW Australia
4. The University of Queensland, St Lucia 4072 QLD Australia
5. VinUni Big Data Research Institute VinUniversity, Hanoi, Vietnam

*Correspondence: Prof Robert Henry (R.J.H.) [robert.henry@uq.edu.au](mailto:robert.henry@uq.edu.au)

**Supplementary Figures (S1-S34)**


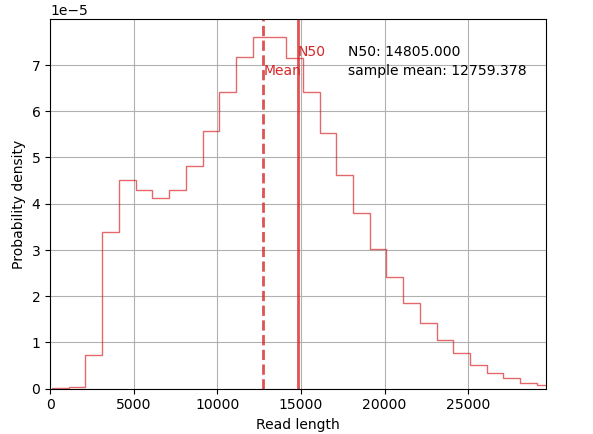


**Figure S1** Length distribution of the PacBio HiFi Reads (sequenced by Sequel II, approximately 27Gb data with approximately 42x coverage for estimated genome size of 650 Mb. (Figure generated by Long QC).


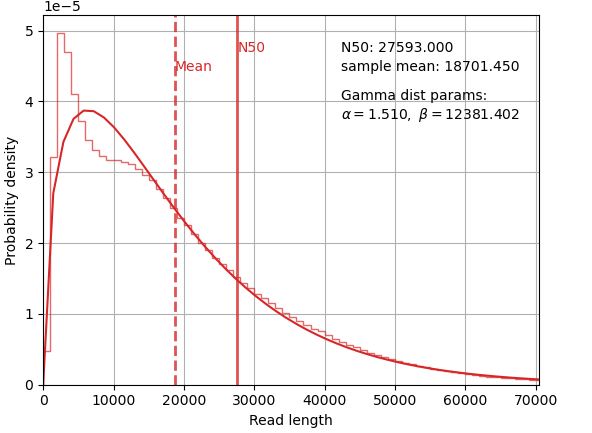


**Figure S2** Length distribution of the ONT Reads (sequenced on Promethion and base called with Guppy basecaller, approximately 127 Gb data at approximately 195x coverage for estimated genome size of 650 Mb. (Figure generated by LongQC).


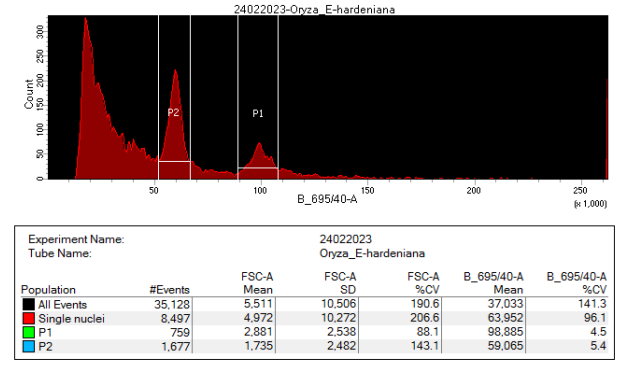


**Figure S3:** **Flow cytometric analysis for genome size estimation of** Eidothea hardeniana **using** Oryza sativa **as a reference standard.** The histogram (x-axis: fluorescence intensity in channel B_695/40-A; y-axis: event count) shows distinct peaks corresponding to different nuclei populations (P1 *Eidothea hardeniana*, P2 *Oryza sativa*). The table below provides the mean forward scatter (FSC-A), standard deviation (SD), coefficient of variation (%CV), and mean fluorescence (B_695/40-A Mean) for each gated population. By comparing the peak positions of P1 with the reference P2, the relative genome size of E. hardeniana was inferred.

*
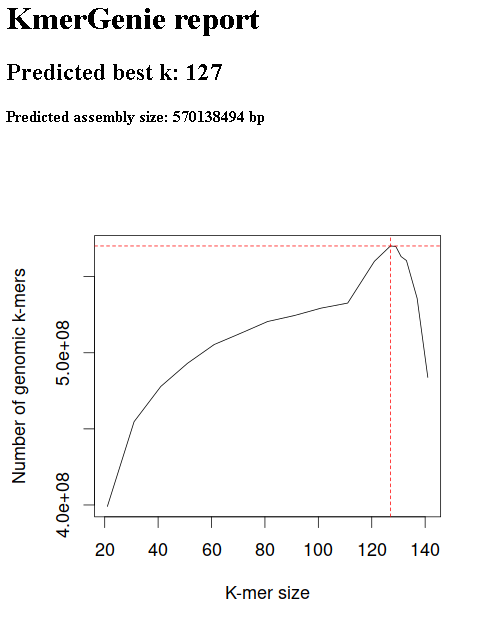
*

**Figure S4** Genome assembly size estimation using Kmergenie best predicted k-mer size 127.

*
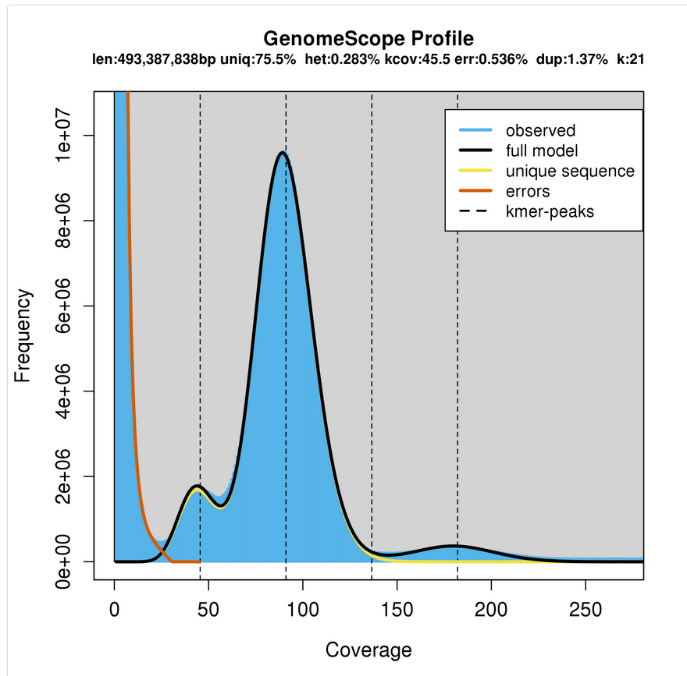
*

**Figure S5** Genome size and heterozygosity estimation using GenomeScope and k-mer size 21.


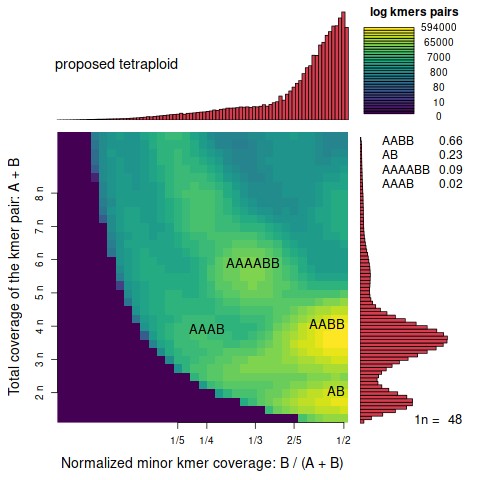


**Figure S6** Smudge plot analysis of *Eidothea hardeniana* using 21 k‑mer length suggesting a tetraploid genome**.** The central heatmap visualizes the distribution of k‑mer pairs (A, B) based on two axes. The x‑axis (horizontal) shows the *normalized minor k‑mer coverage*, B/(A + B), indicating the proportion of the smaller of the two k‑mers relative to their combined coverage. The y‑axis (vertical) represents the *total coverage* of each k‑mer pair, (A + B). Warmer (yellow) regions denote higher densities of k‑mer pairs, as indicated by the color scale on the top right (log-transformed counts). The histograms on the top and right display marginal distributions for the x‑axis and y‑axis, respectively. Labelled clusters (e.g., “AB,” “AABB,” “AAABB,” “AAAABB”) reflect distinct coverage relationships that, in combination, suggest a tetraploid configuration. The proportions (e.g., 0.66 for AABB) indicate the approximate fraction of k‑mer pairs falling into each category. Together, these patterns support the interpretation that *E. hardeniana* is predominantly tetraploid (proposed 4n).


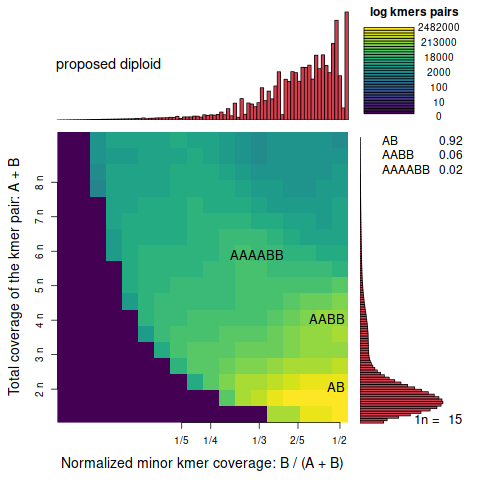


**Figure S7.** Smudgeplot analysis of *Eidothea hardeniana* 127 k‑mer length suggesting a diploid genome. This diagram visualizes the distribution of paired k‑mers (A, B) along two dimensions. The x‑axis (horizontal) represents the *normalized minor k‑mer coverage*, B/(A + B), indicating the fraction of the smaller k‑mer relative to the total coverage of both k‑mers. And the y‑axis represents the *total coverage* of the pair, (A + B). Color intensity (yellow to dark blue) corresponds to the log‑transformed count of k‑mer pairs (top‑right color scale), with warmer colors denoting higher densities. Marginal histograms above and to the right depict the distribution of values along each axis. Clusters labeled “AB,” “AABB,” and “AAAABB” reflect different coverage relationships. In this sample, the dominant “AB” cluster (~0.92) supports a primarily diploid (2n) genome configuration.

**Figure S8:** The comparison of the number of contigs in assemblies of *E. hardeniana* when generated using different combinations of the sequencing data. First assembly was generated suing only hifi and hi-c reads, subsequently whole ONT data and length based filtered ONT reads (>20Kb, >30kb, >40kb and >50kb) were used to generate assemblies with hifiasm.

**Figure S9:** Comparison of the number of T2T scaffolds obtained after scaffolding with Yahs


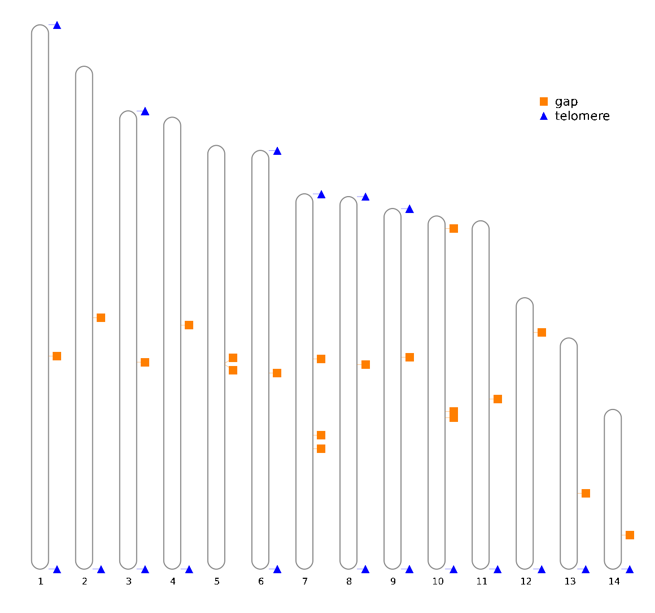


**Figure S10: Telomeric repeats and assembly gaps across the 14 longest chromosome-scale scaffolds of the** Eidothea hardeniana **hybrid assembly (HiFi + Hi-C).**Each vertical bar represents one pseudochromosome (1–14, ordered by decreasing length). Blue triangles indicate positions of telomeric repeat arrays, and orange squares mark internal assembly gaps (stretches of ambiguous bases, Ns) along each scaffold.


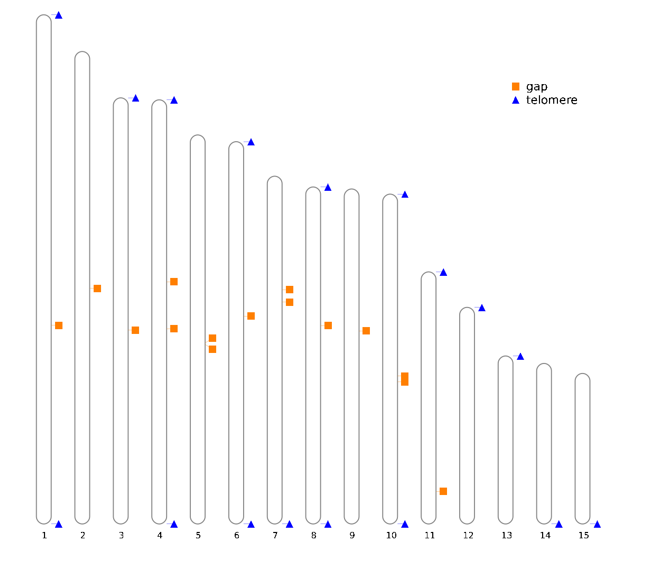


**Figure S11: Telomeric repeats and assembly gaps across the 14 longest chromosome-scale scaffolds of the** Eidothea hardeniana **hybrid assembly (HiFi + ONT (unfiltered) + Hi-C).**Each vertical bar represents one pseudochromosome (1–14, ordered by decreasing length). Blue triangles indicate positions of telomeric repeat arrays, and orange squares mark internal assembly gaps (stretches of ambiguous bases, Ns) along each scaffold.


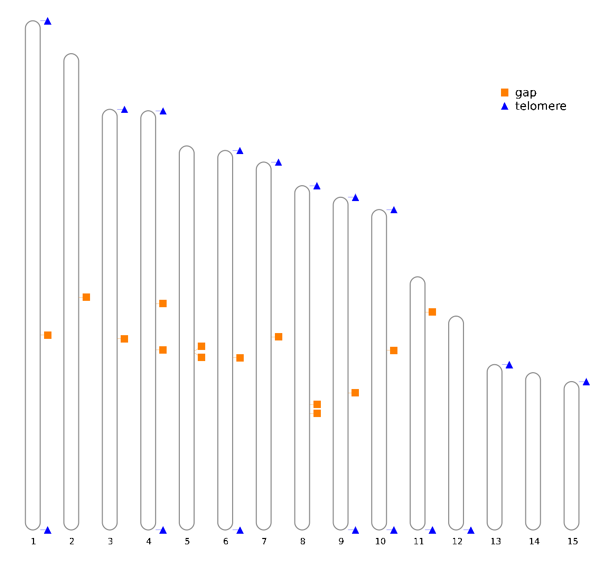


**Figure S12: Telomeric repeats and assembly gaps across the 14 longest chromosome-scale scaffolds of the** Eidothea hardeniana **hybrid assembly (HiFi + ONT ≥ 20 kb + Hi-C).**Each vertical bar represents one pseudochromosome (1–14, ordered by decreasing length). Blue triangles indicate positions of telomeric repeat arrays, and orange squares mark internal assembly gaps (stretches of ambiguous bases, Ns) along each scaffold.


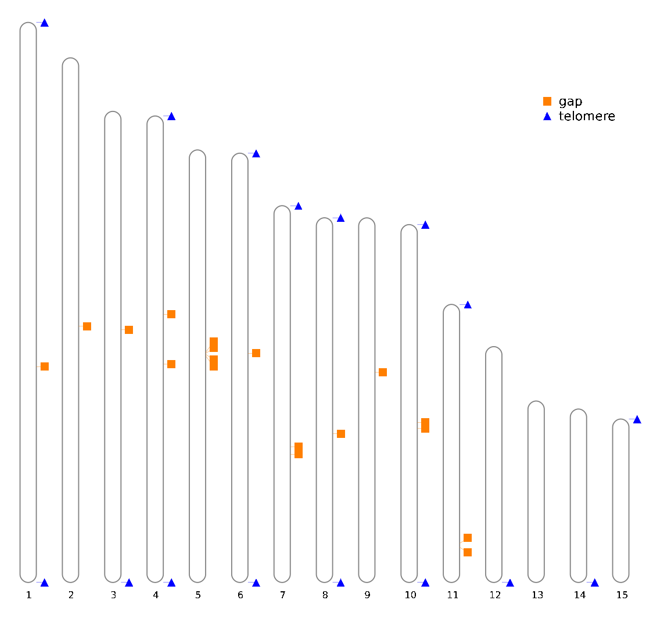


**Figure S13: Telomeric repeats and assembly gaps across the 14 longest chromosome-scale scaffolds of the** Eidothea hardeniana **hybrid assembly (HiFi + ONT ≥ 30 kb + Hi-C).**Each vertical bar represents one pseudochromosome (1–14, ordered by decreasing length). Blue triangles indicate positions of telomeric repeat arrays, and orange squares mark internal assembly gaps (stretches of ambiguous bases, Ns) along each scaffold.


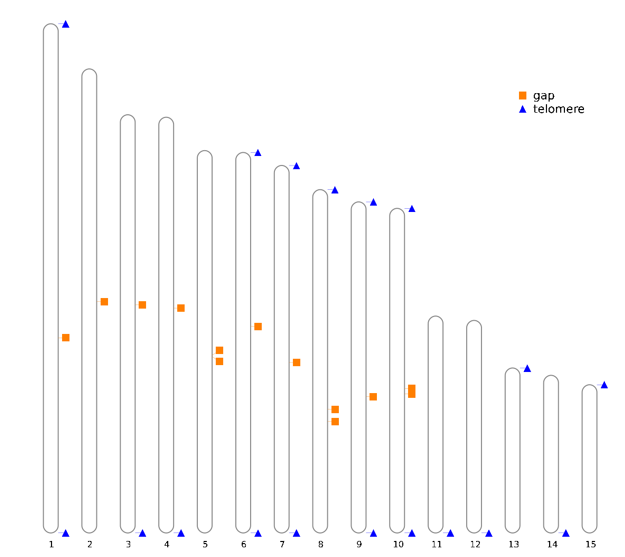


**Figure S14: Telomeric repeats and assembly gaps across the 14 longest chromosome-scale scaffolds of the** Eidothea hardeniana **hybrid assembly (HiFi + ONT ≥ 40 kb + Hi-C).**Each vertical bar represents one pseudochromosome (1–14, ordered by decreasing length). Blue triangles indicate positions of telomeric repeat arrays, and orange squares mark internal assembly gaps (stretches of ambiguous bases, Ns) along each scaffold.


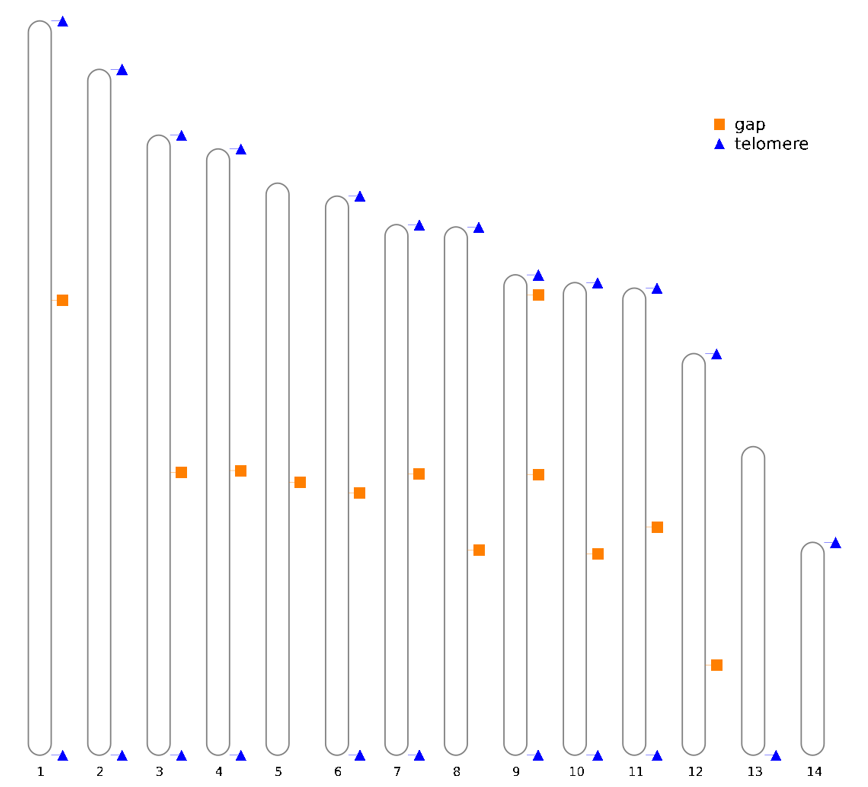


**Figure** **S15.** **Telomeric repeats and assembly gaps across the 14 longest chromosome-scale scaffolds of the** Eidothea hardeniana **hybrid assembly (HiFi + ONT ≥ 50 kb + Hi-C).**Each vertical bar represents one pseudochromosome (1–14, ordered by decreasing length). Blue triangles indicate positions of telomeric repeat arrays, and orange squares mark internal assembly gaps (stretches of ambiguous bases, Ns) along each scaffold.


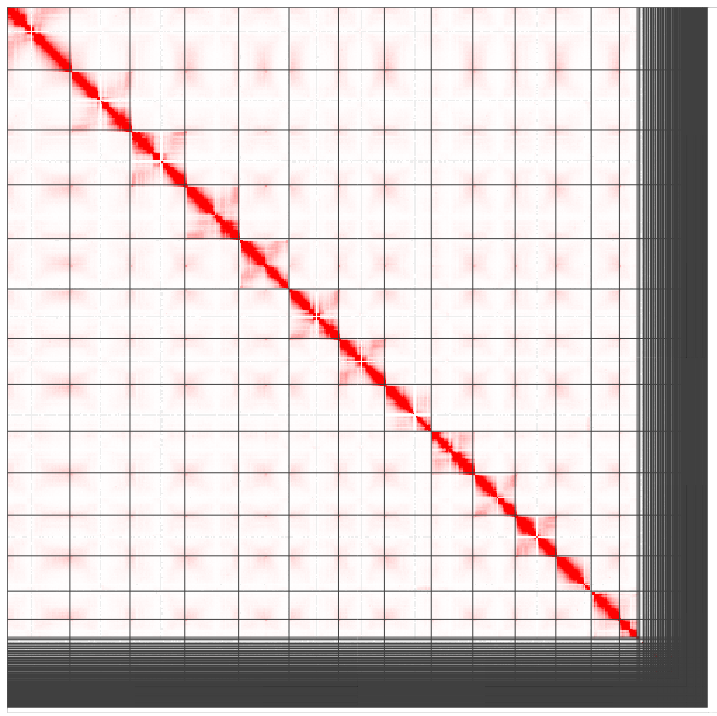


**Figure S16: Genome-wide Hi-C contact map of the** Eidothea hardeniana **assembly produced by YaHS.** Hi-C interaction frequencies are shown as a heatmap for all chromosome-scale scaffolds ordered along both axes. The strong, continuous main diagonal indicates high contact frequency among neighbouring loci and supports the global contiguity and ordering of the 12 chromosome-scale scaffolds, while the grid lines mark scaffold boundaries. Off-diagonal contacts are relatively weak and sparse, consistent with limited inter-chromosomal mis-joins in the final assembly.


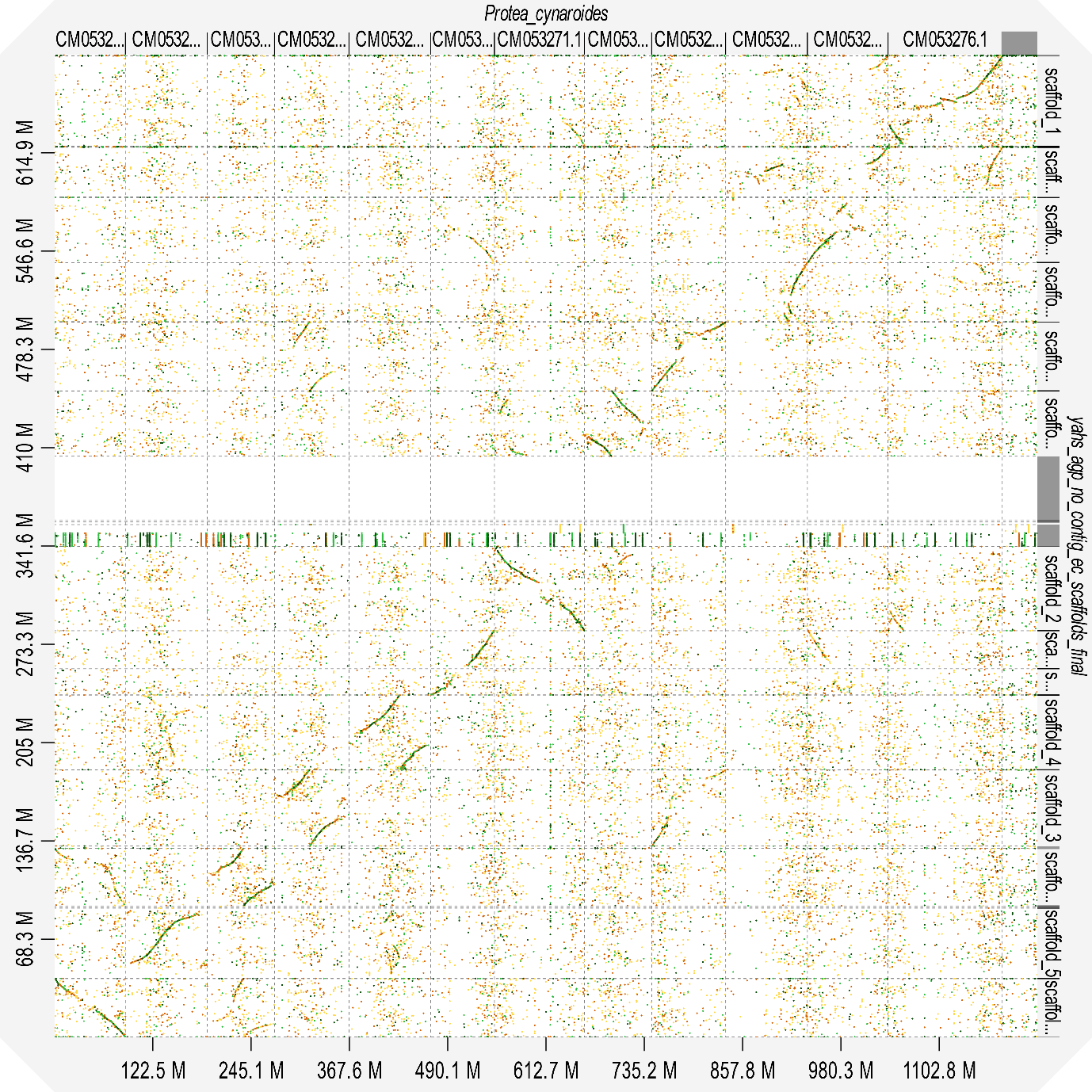


**Figure S17** *Eidothea* assembly with 14 chromosome scale scaffolds aligned against *Protea* *cynaroides*.


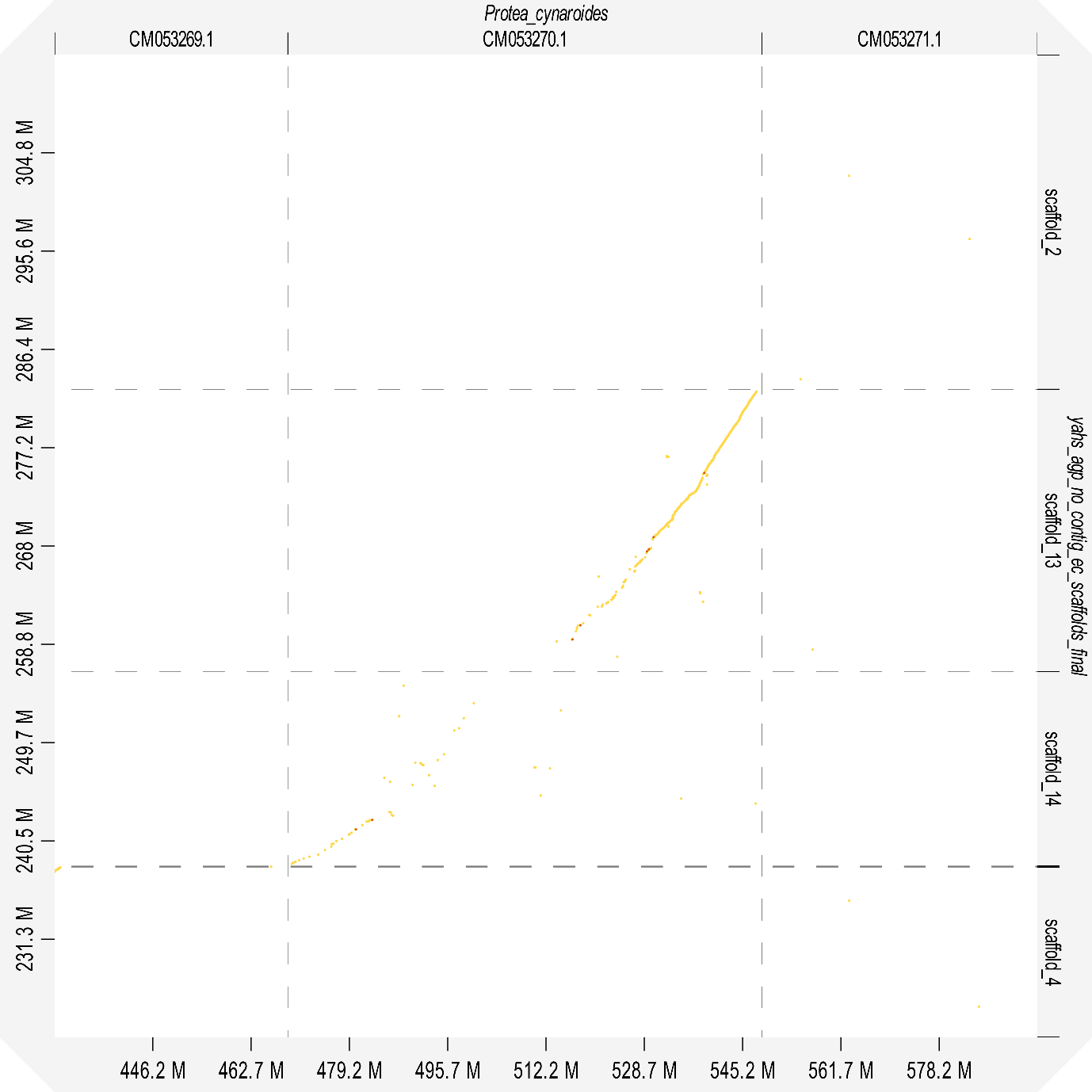


**Figure S18:** Scaffold 13 and scaffold 14 of the *Eidothea* assembly showing high collinearity with the chromosome 6 of *Protea cynaroides*.


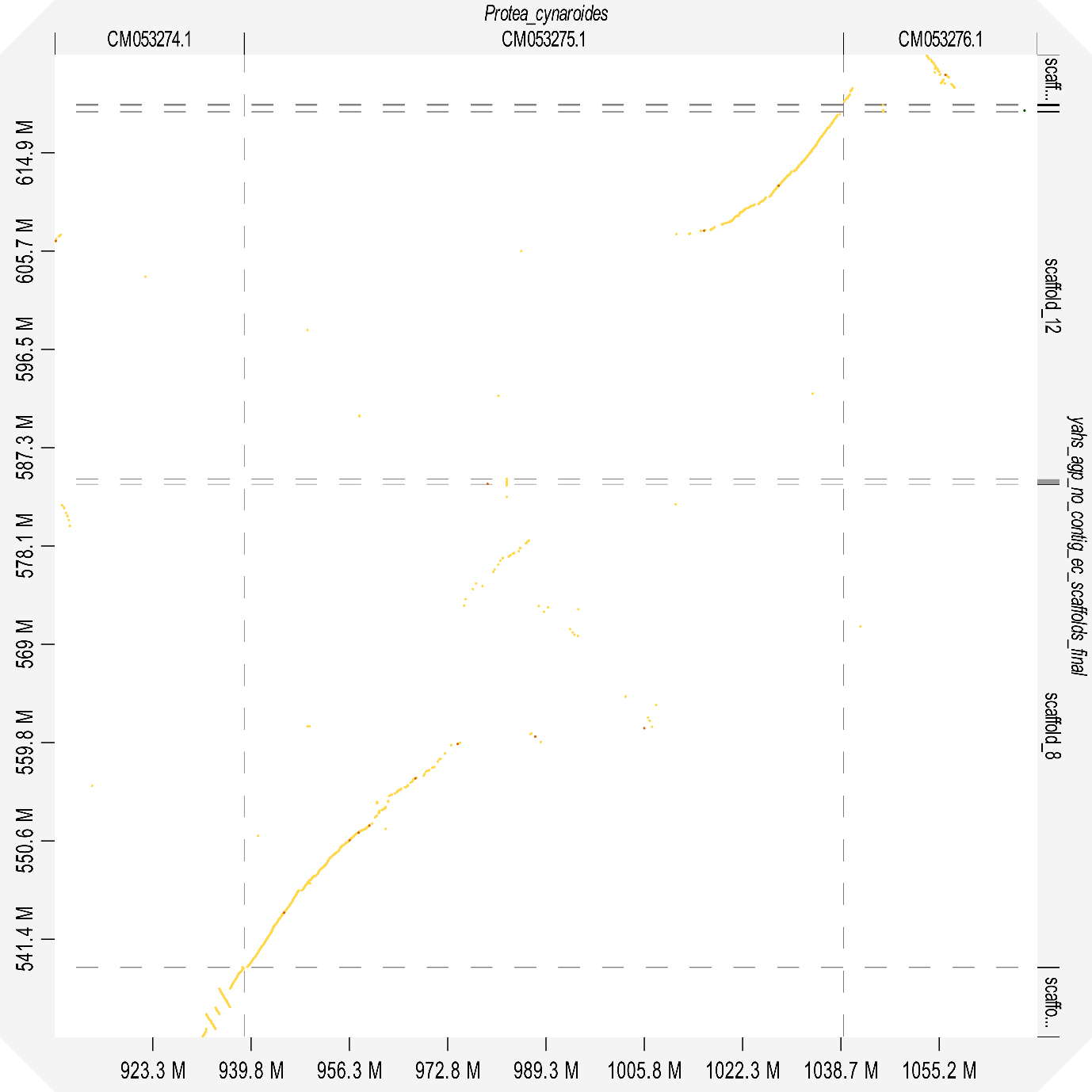


**Figure S19:** Scaffold 8 and scaffold 12 of the *Eidothea hardeniana* assembly showing high collinearity with the chromosome 11 of *Protea cynaroides*.


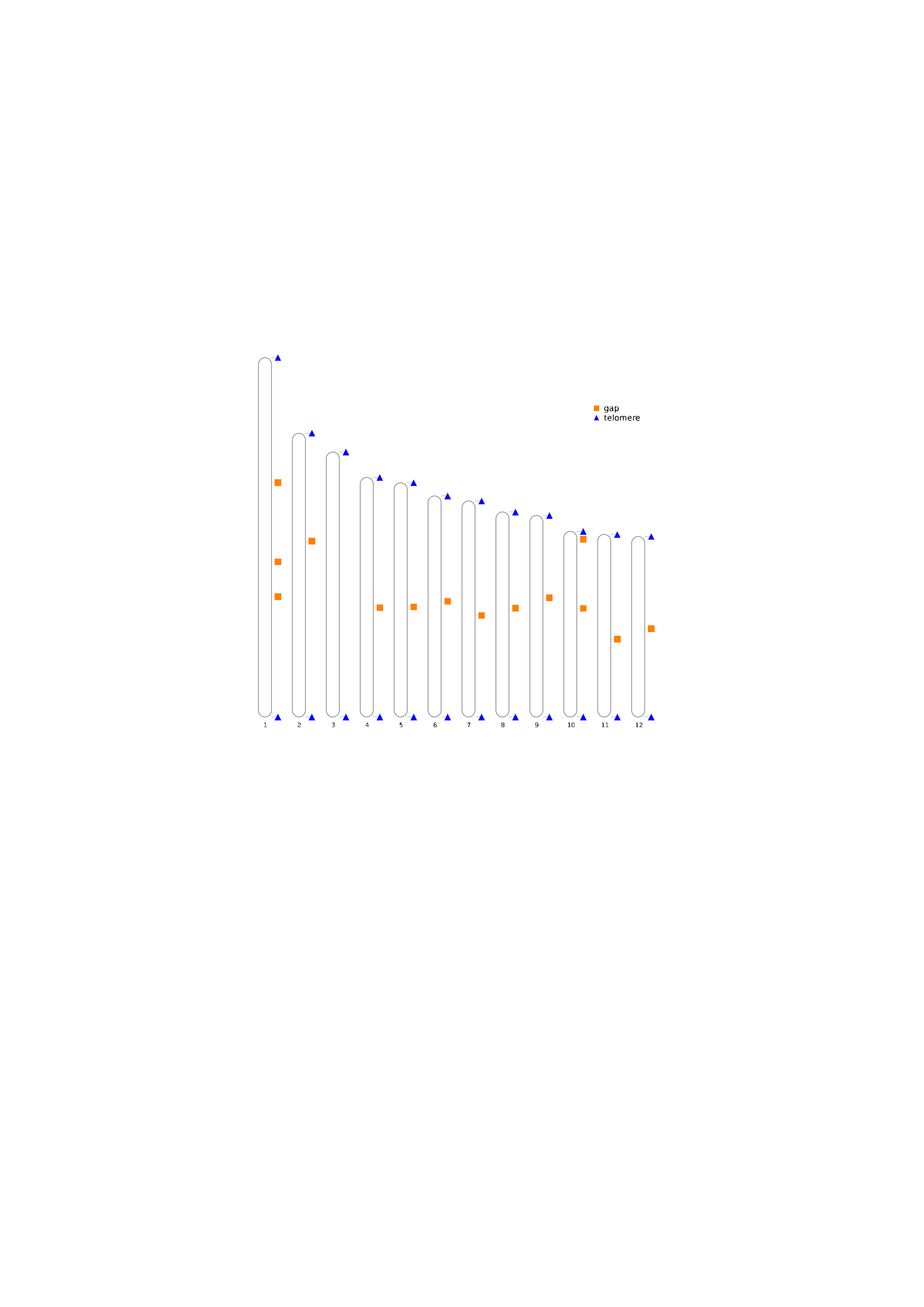
 **Figure** **S20**. **Telomeric repeats and assembly gaps across the 12 longest chromosome-scale scaffolds of the** Eidothea hardeniana **hybrid assembly (HiFi + ONT ≥ 50 kb + Hi-C).**Each vertical bar represents one pseudochromosome (1–12, ordered by decreasing length). Blue triangles indicate positions of telomeric repeat arrays, and orange squares mark internal assembly gaps (stretches of ambiguous bases, Ns) along each scaffold.


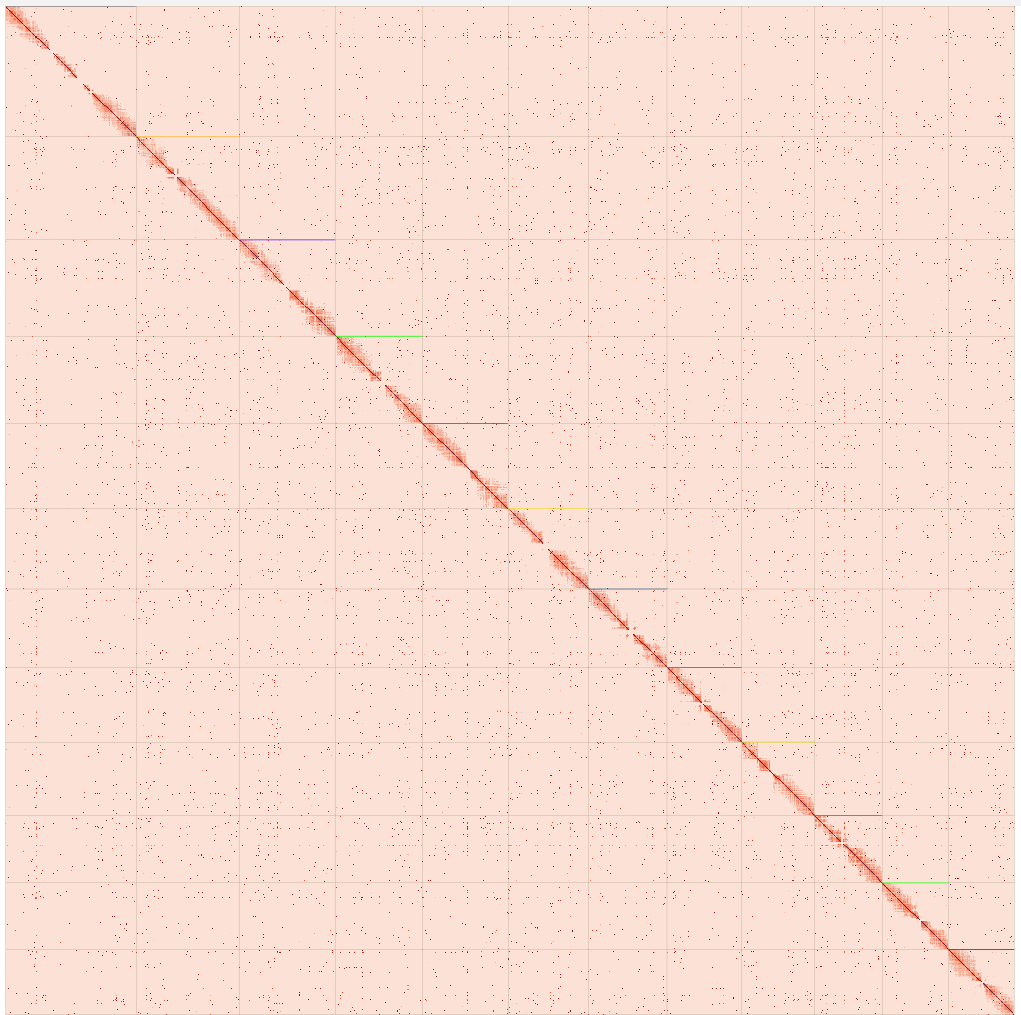


**Figure S21** Hi-C Contact map for the final consensus assembly with 12 pseudochromosomes.

**
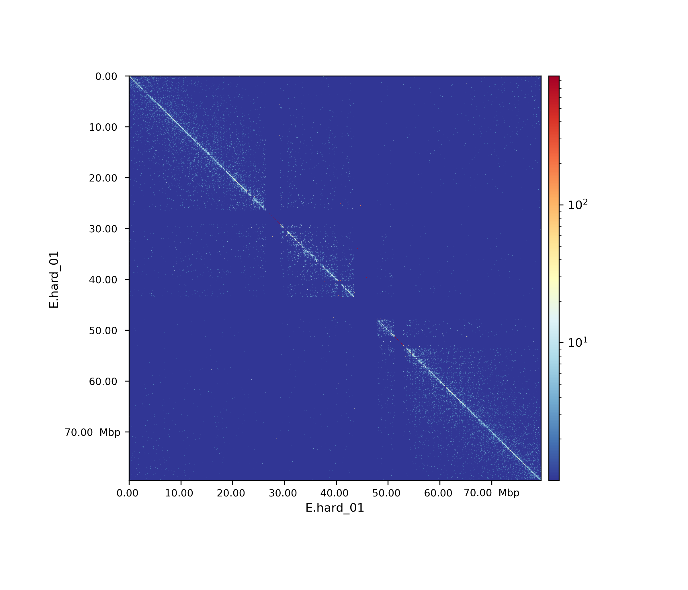

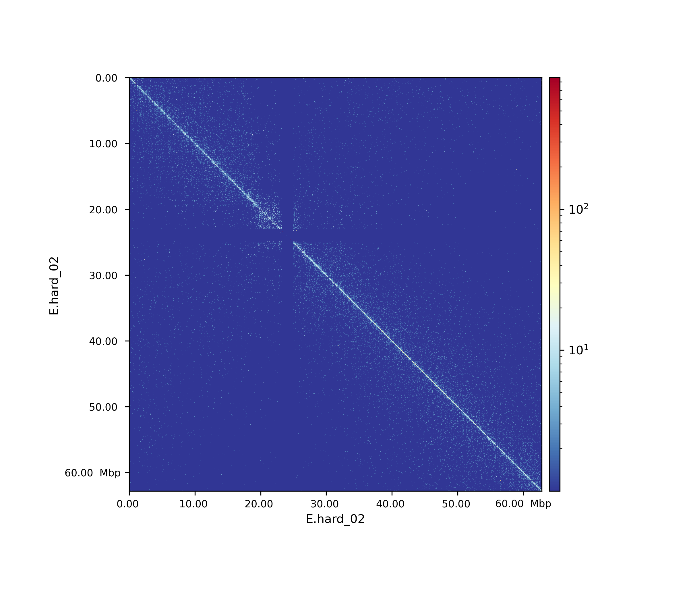

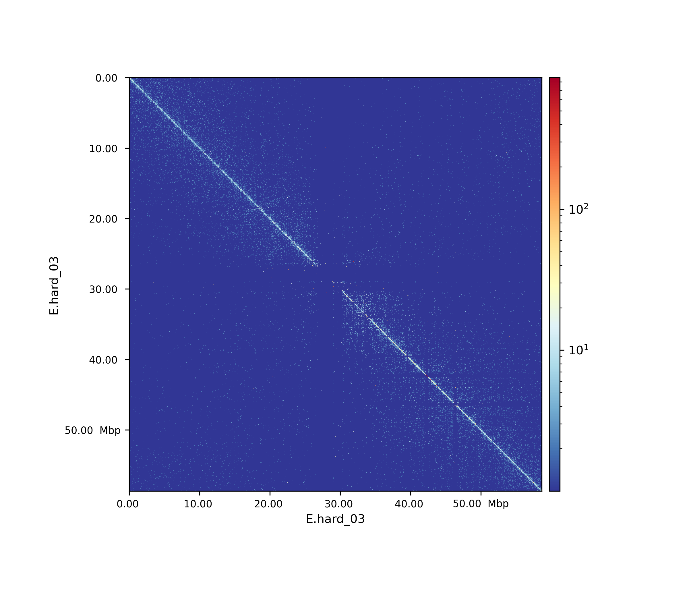

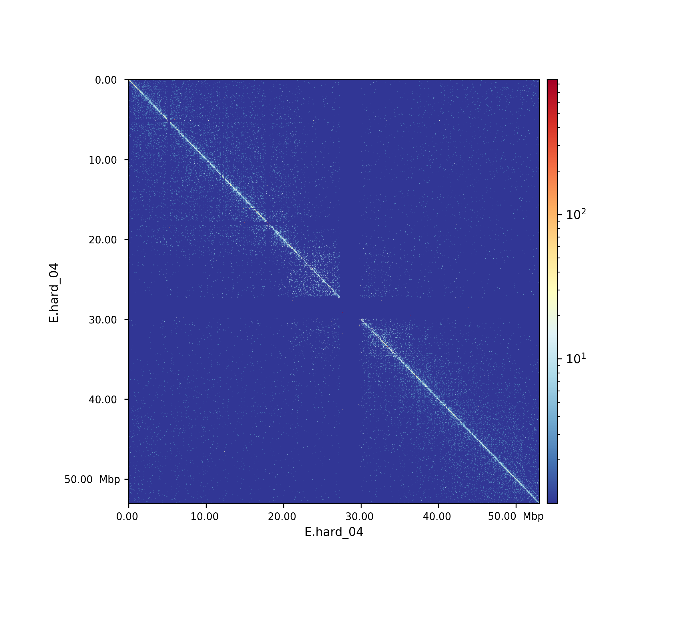

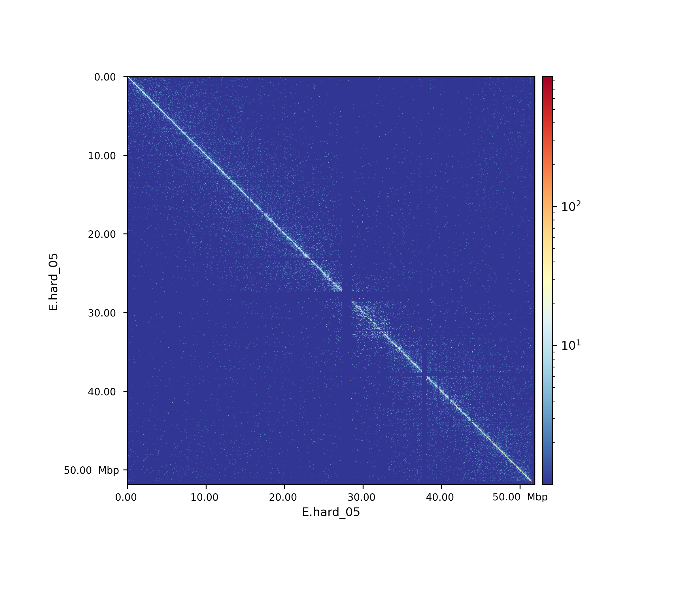

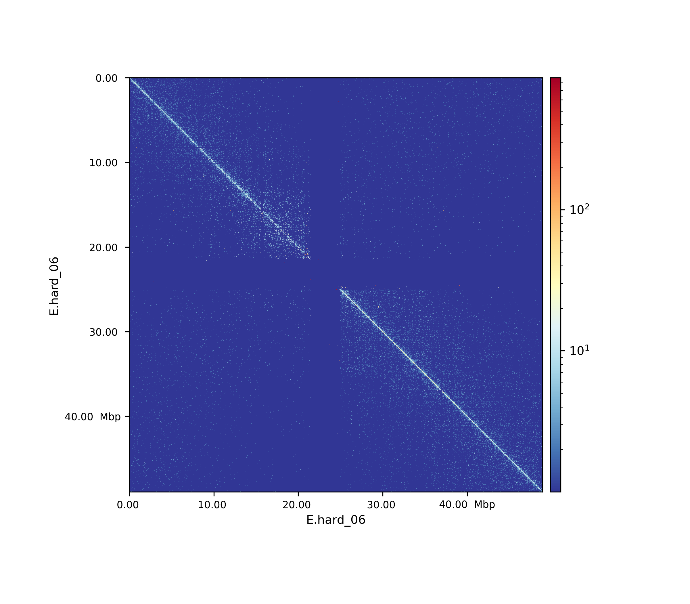

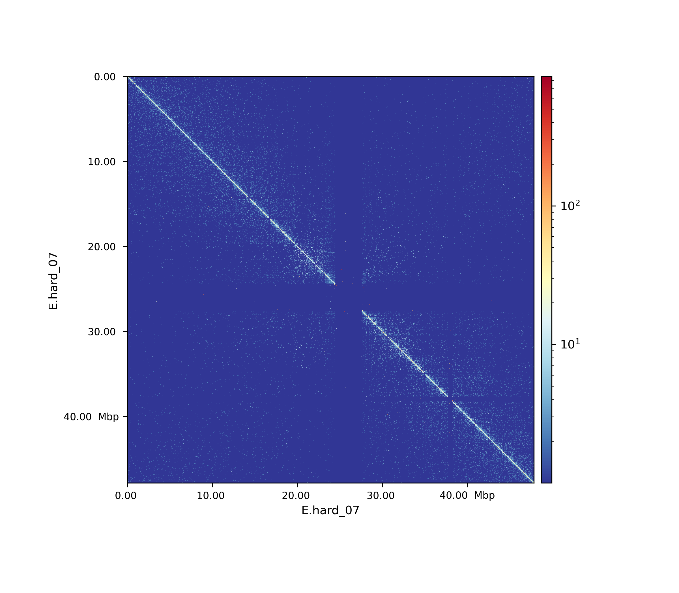

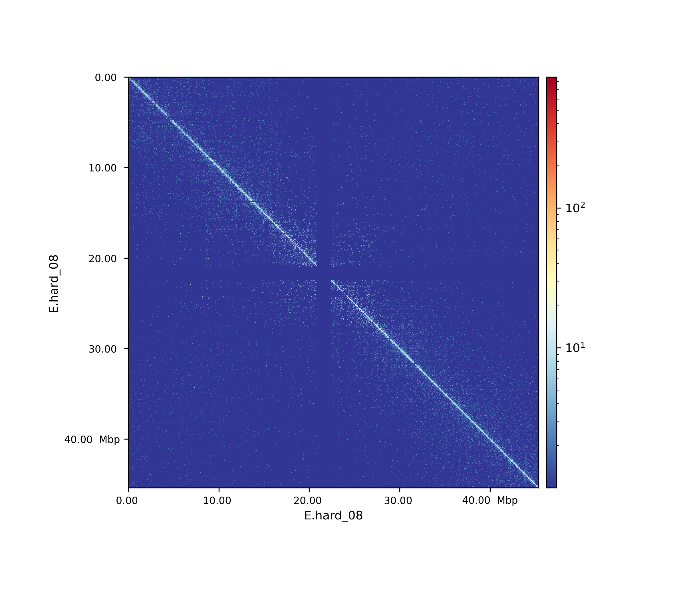

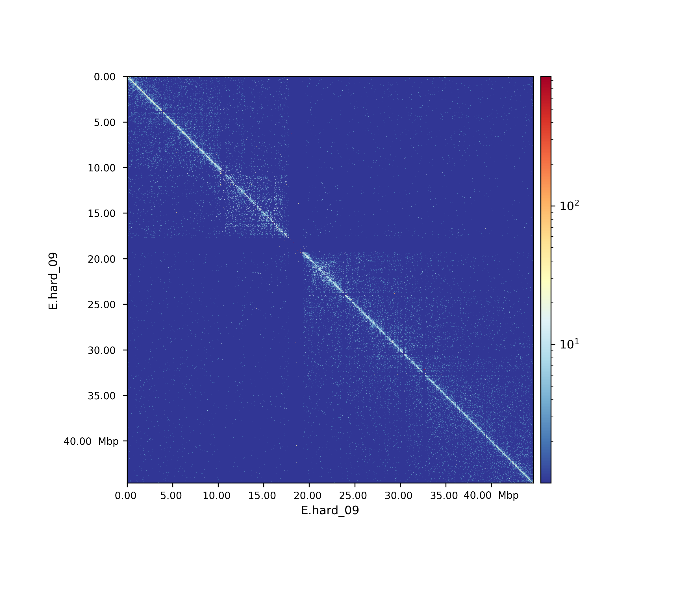

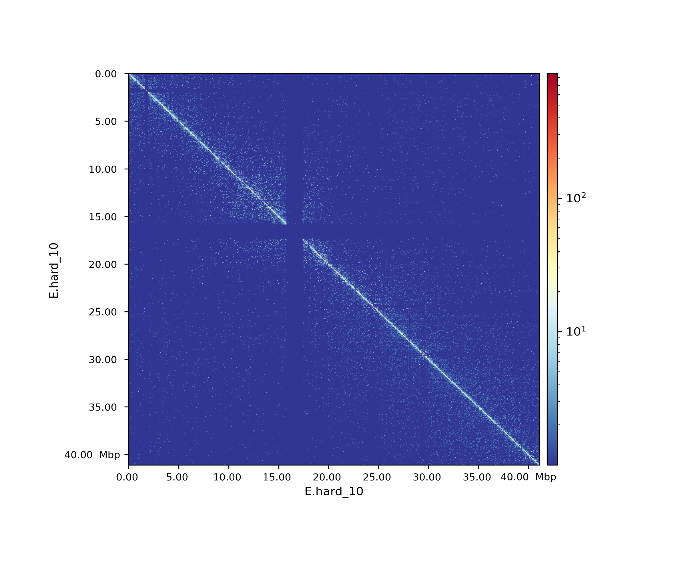

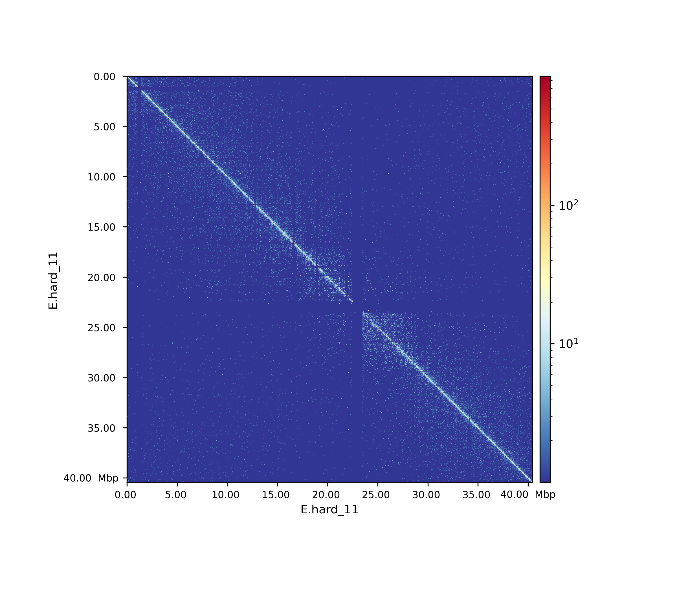

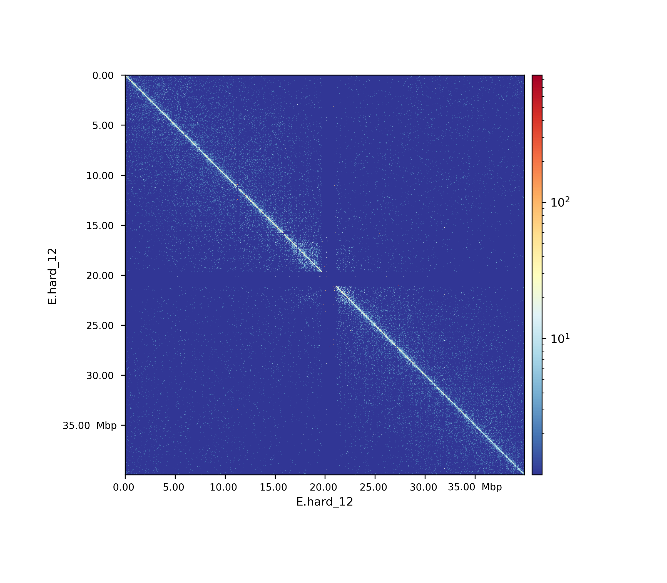
Figure S22 Hi-C contact map for 12 pseudochromosomes of** Eidothea hardeniana**.** Intra-chromosomal Hi-C contact frequencies are shown as a heatmap (log_10_–scaled), with genomic position along on both axes (Mbp). The strong main diagonal indicates high interaction frequency between neighbouring loci and supports the continuity of the scaffold, while the cross-shaped reduction in contacts marks the putative centromeric/pericentromeric region.

**
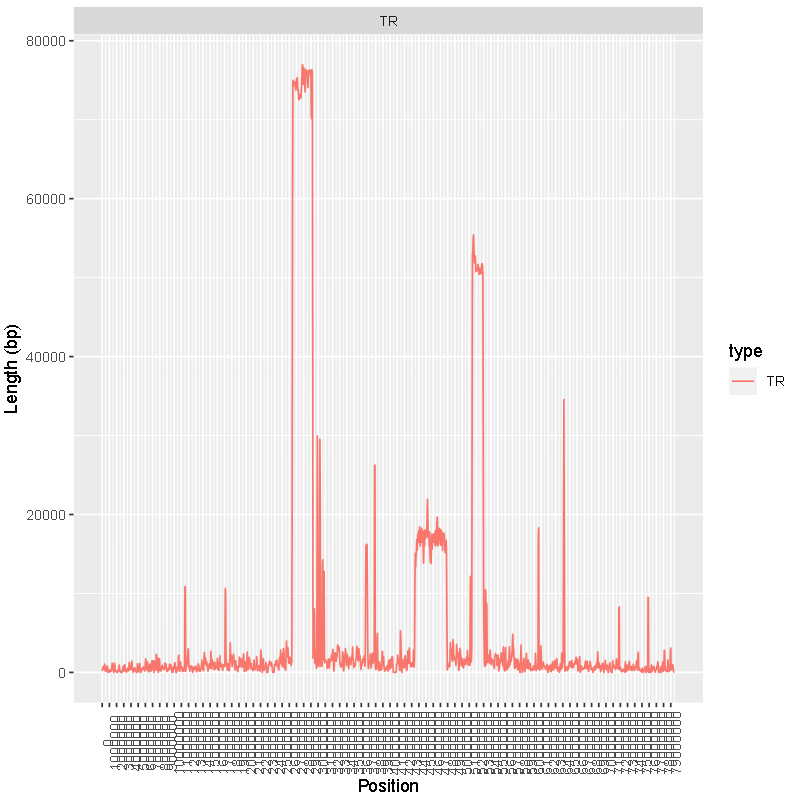
**
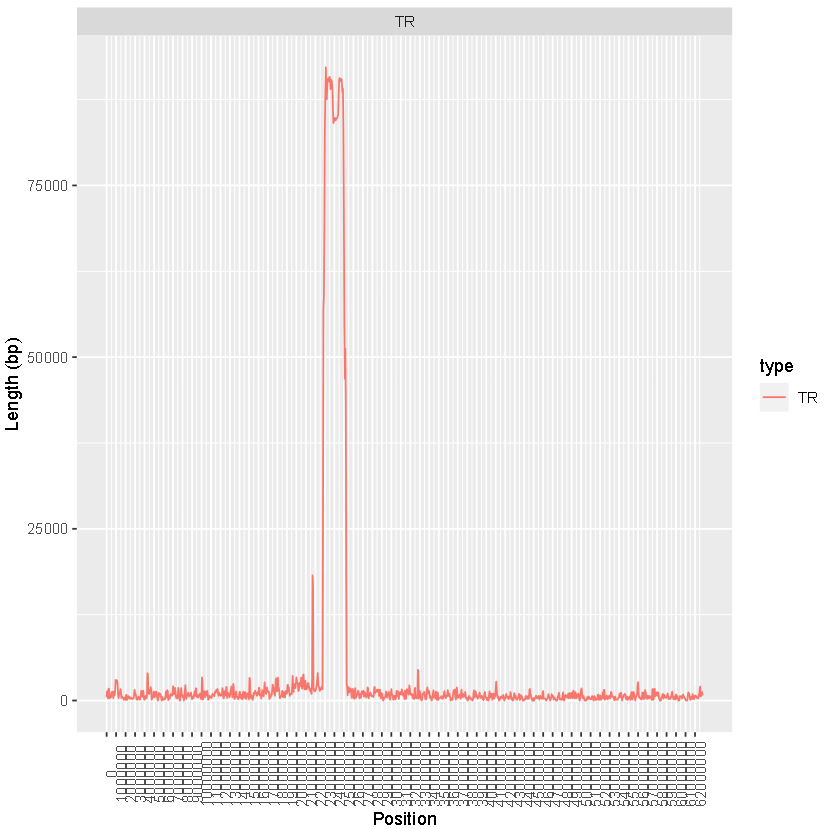


E.hard_02

E.hard_01


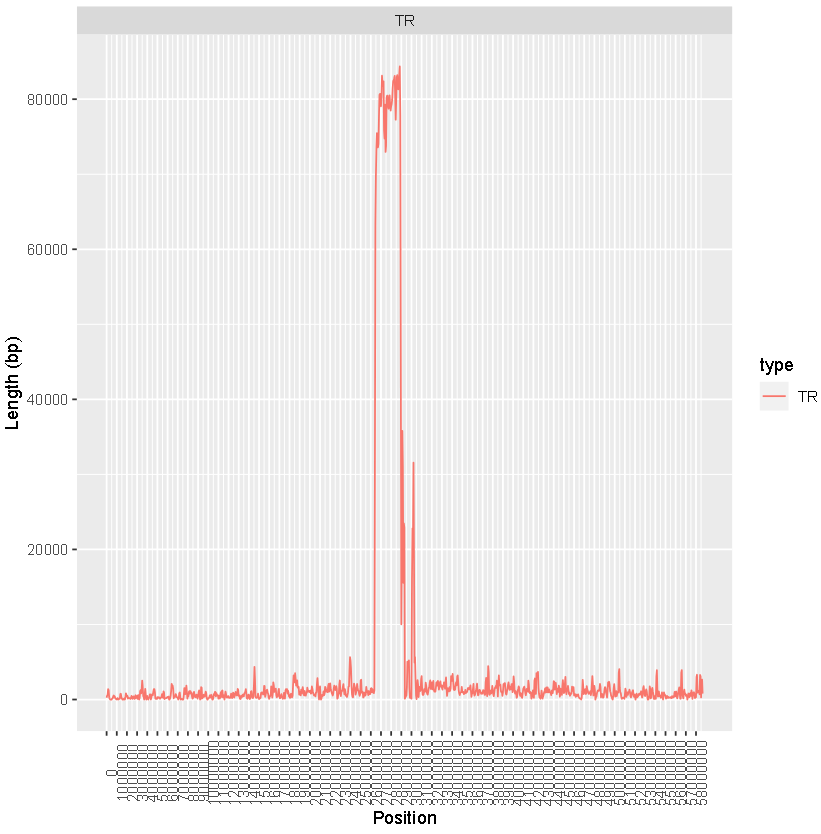

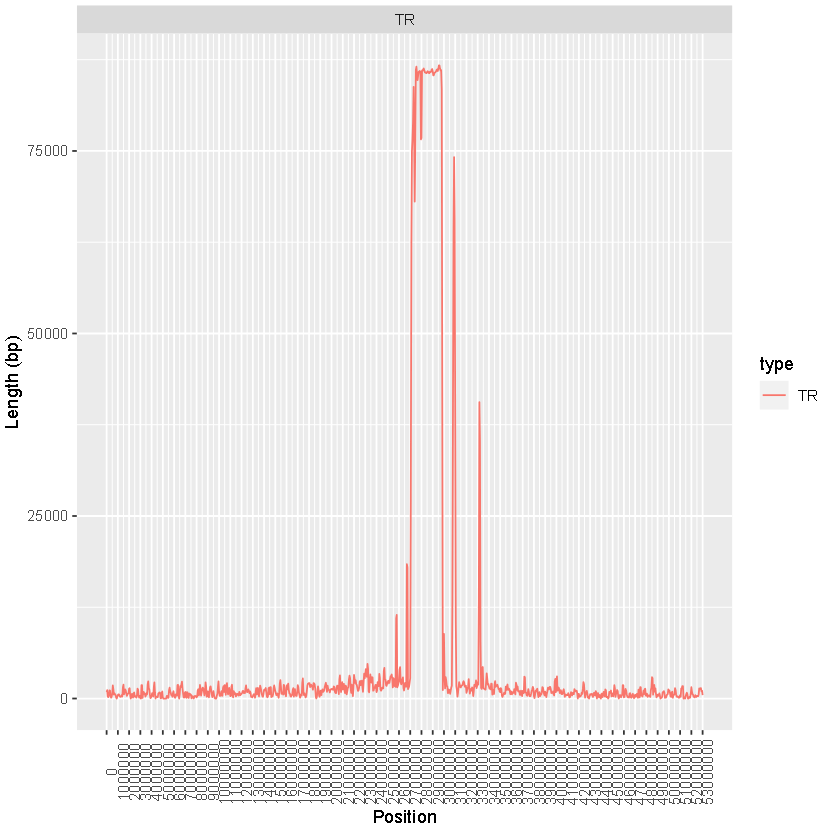


E.hard_04

E.hard_03


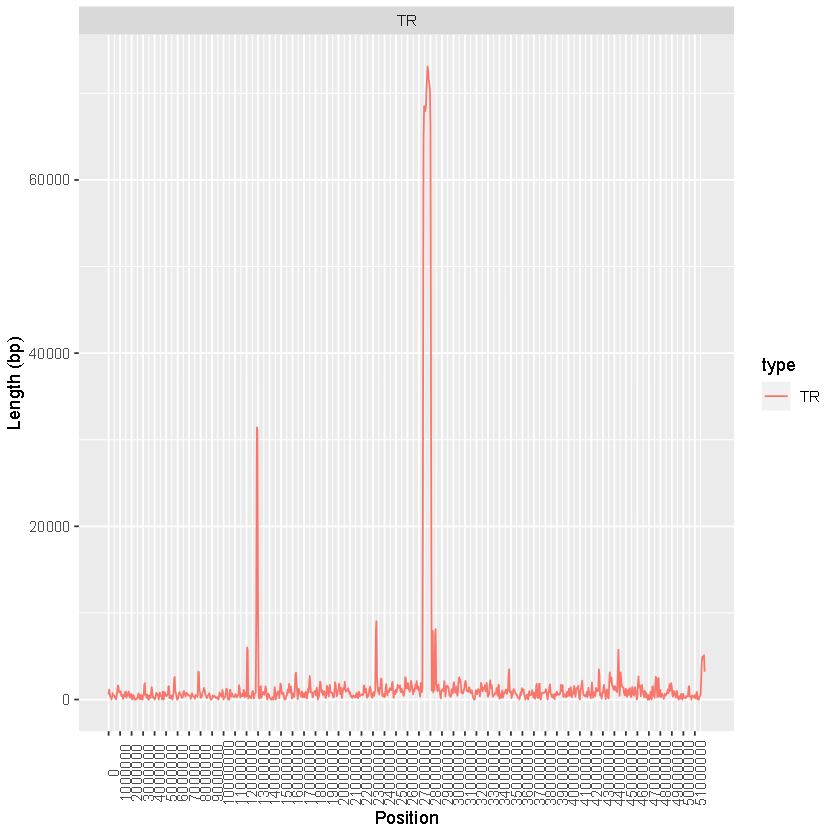

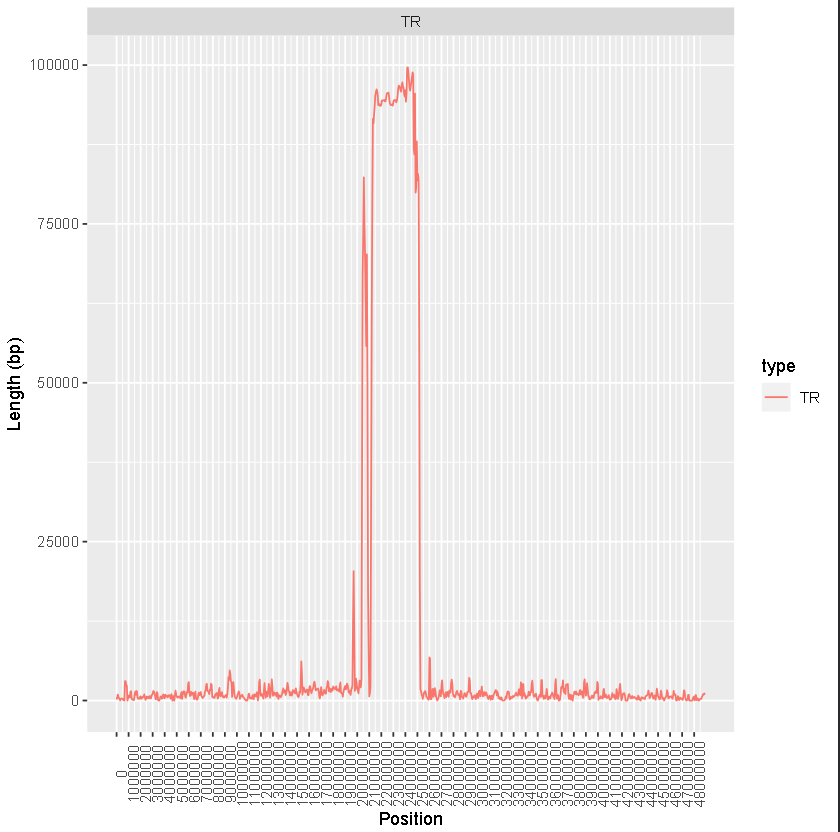


E.hard_05

E.hard_06


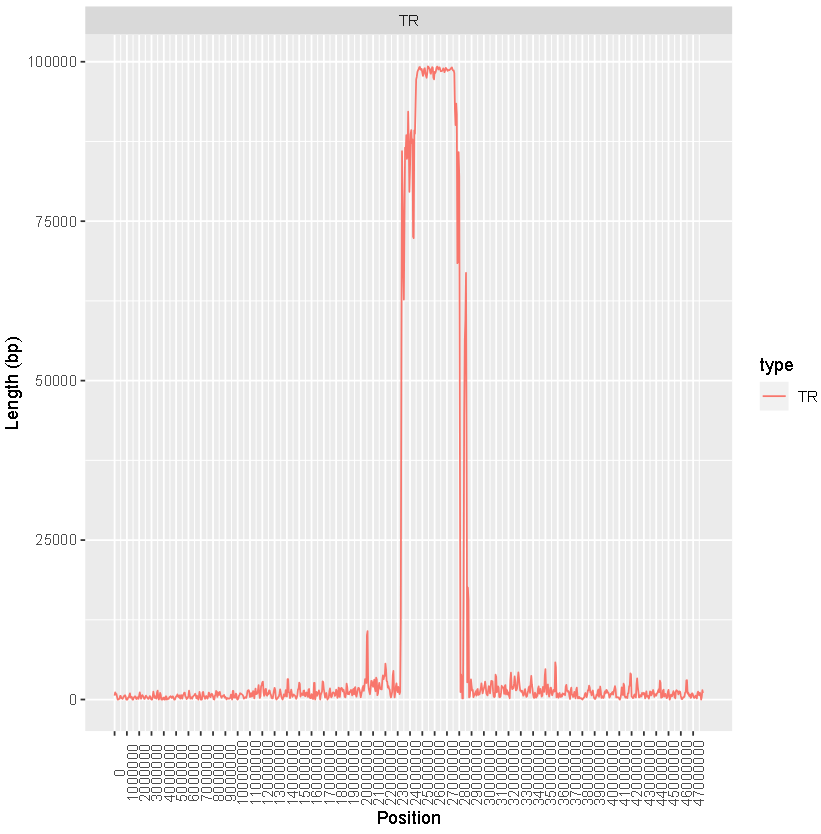

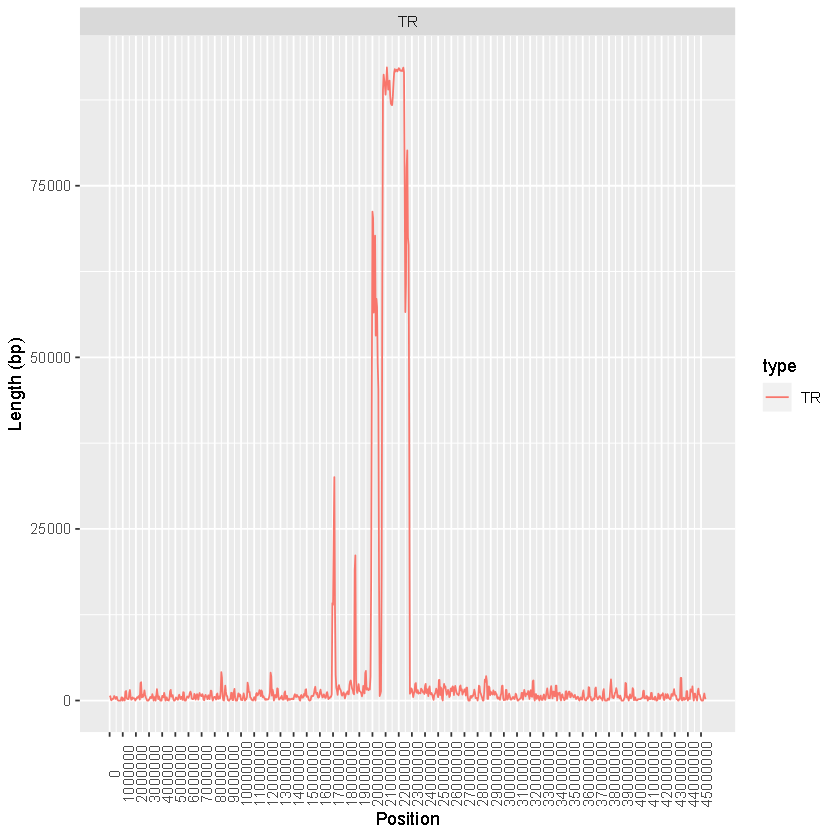


E.hard_07

E.hard_08


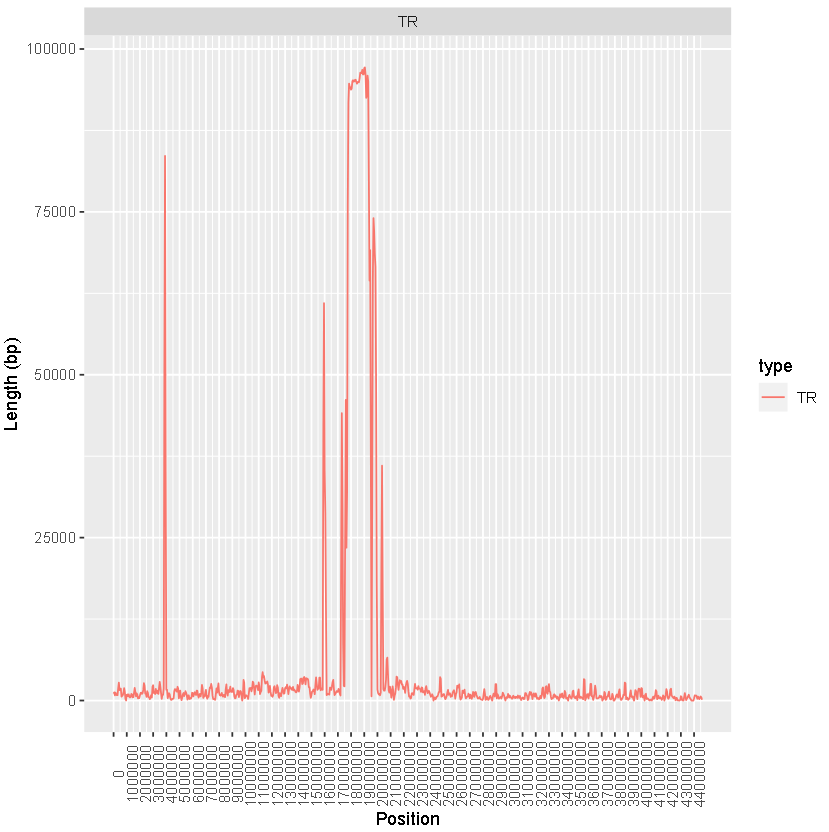

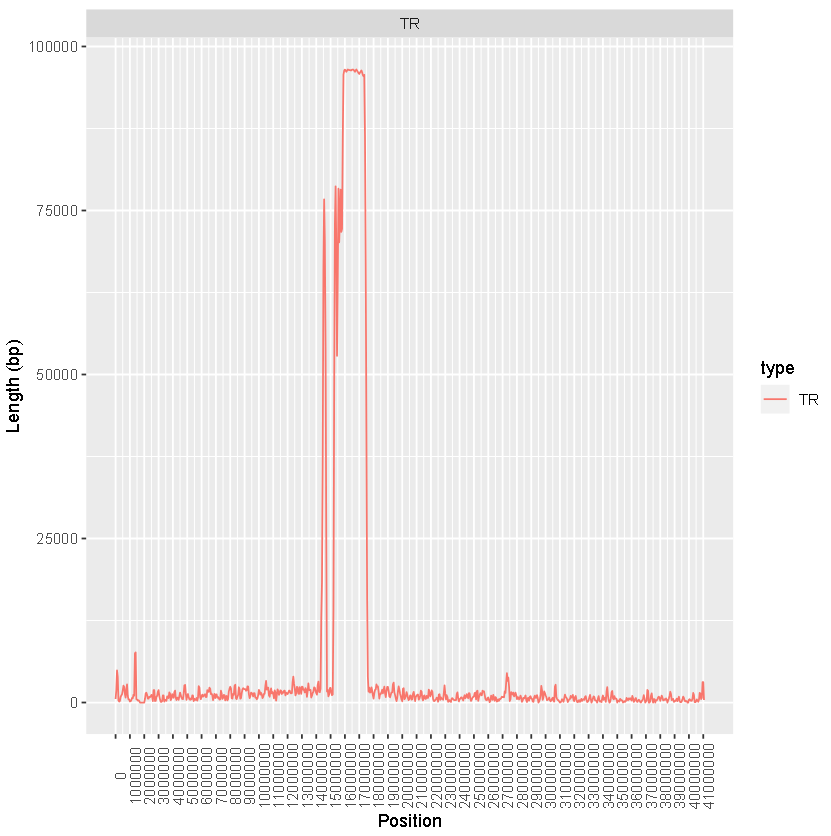


E.hard_09

E.hard_10


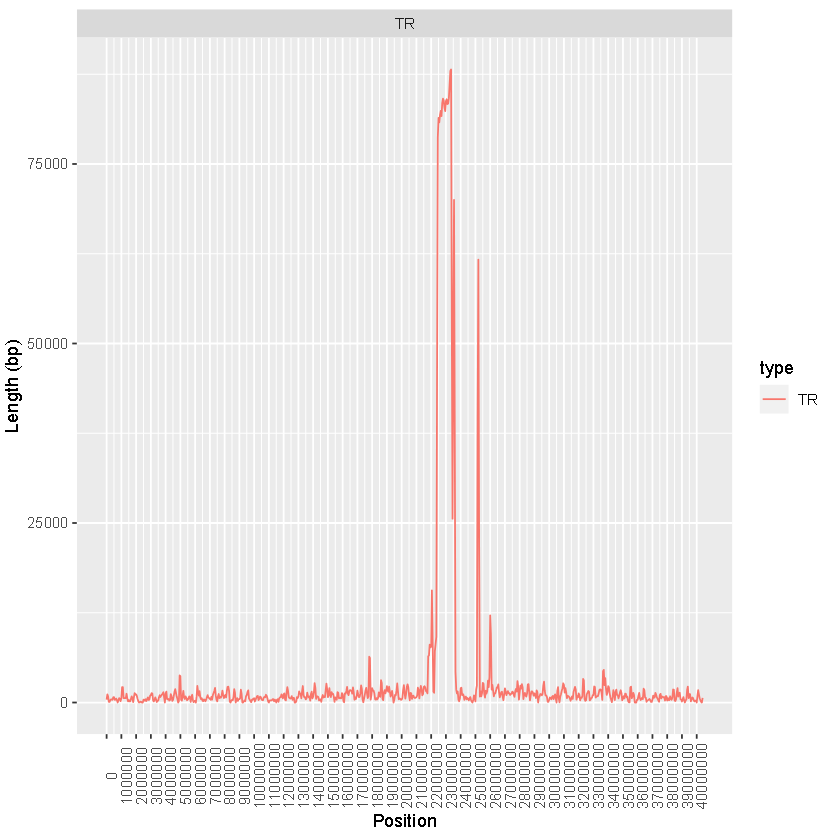

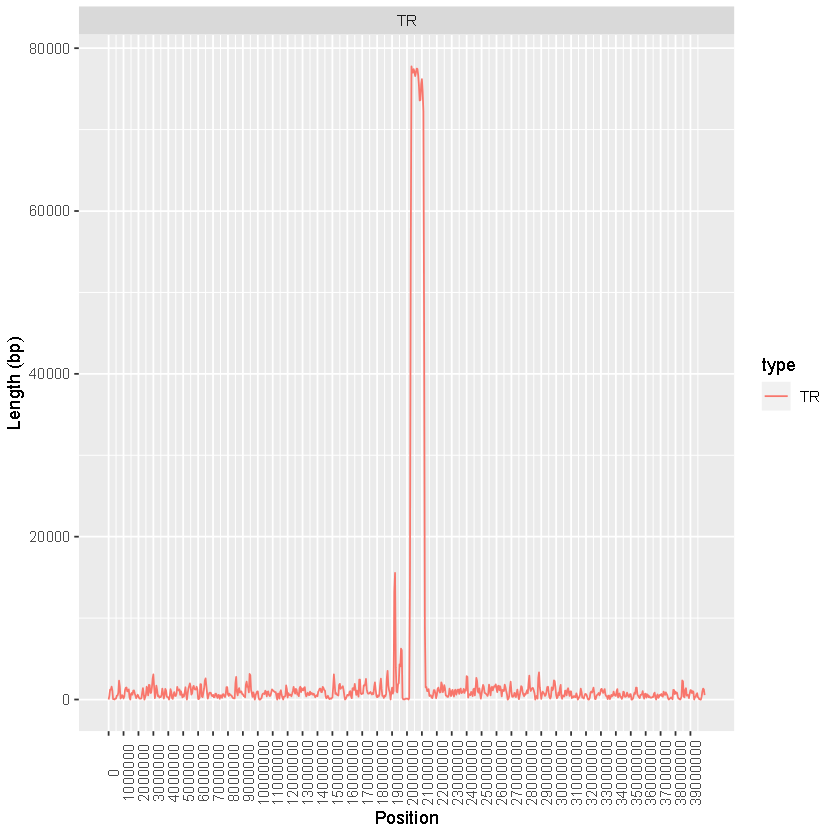


E.hard_11

E.hard_12

**Figure** **S23** **Tandem repeat landscapes along the 12** pseudochromosomes **of** Eidothea hardeniana**.** For each chromosome, the total length (bp) of tandem repeat (TR) sequences within consecutive genomic windows is plotted against physical position along the chromosome. TRs were identified with the QuarTeT/CentroMiner pipeline from the assembled genome sequence. Sharp peaks indicate localized enrichment of tandem repeats and mark the putative centromeric/pericentromeric regions on each chromosome.


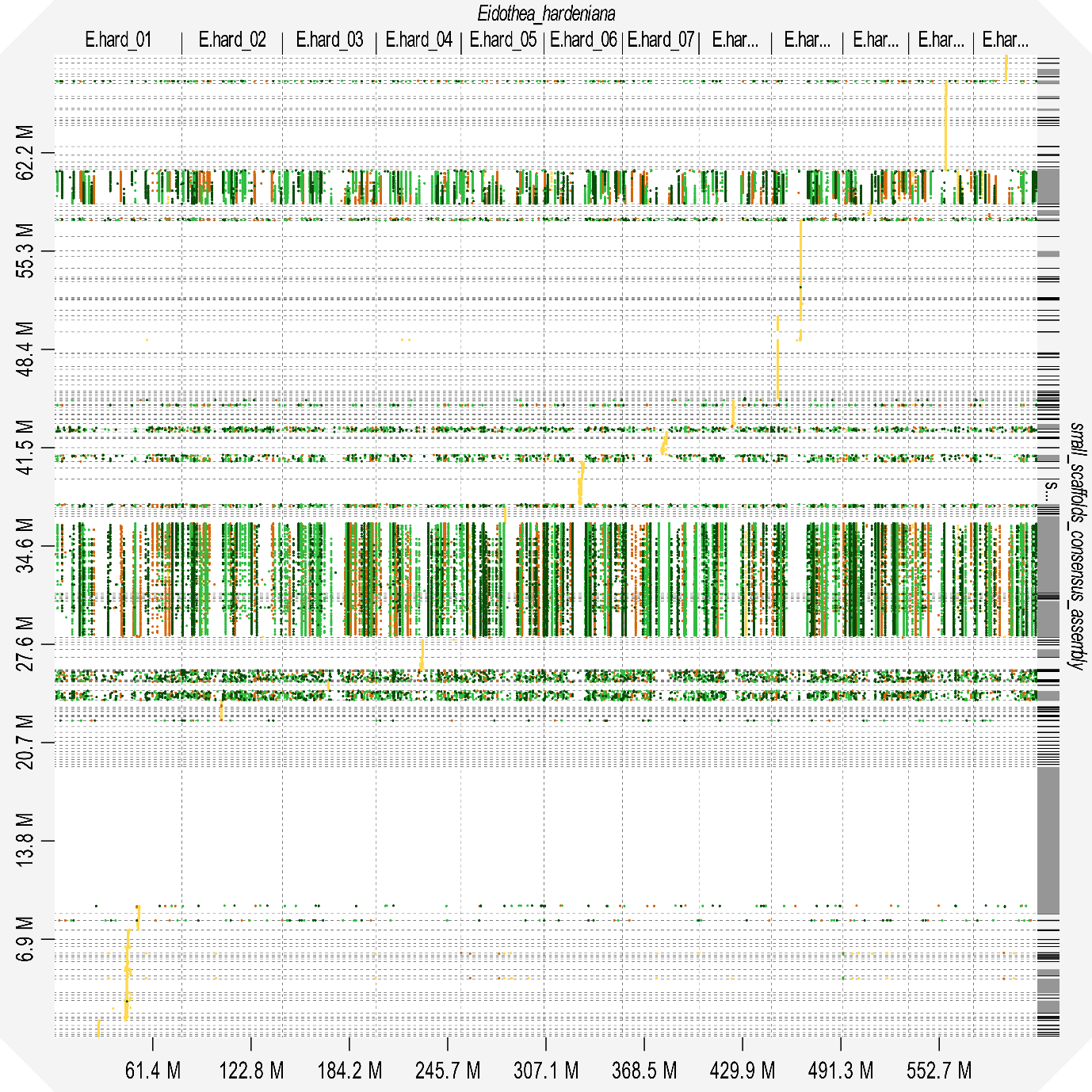


**Figure S24** Small unplaced scaffolds aligned against *Eidothea* assembly with 12 pseudochromosomes.


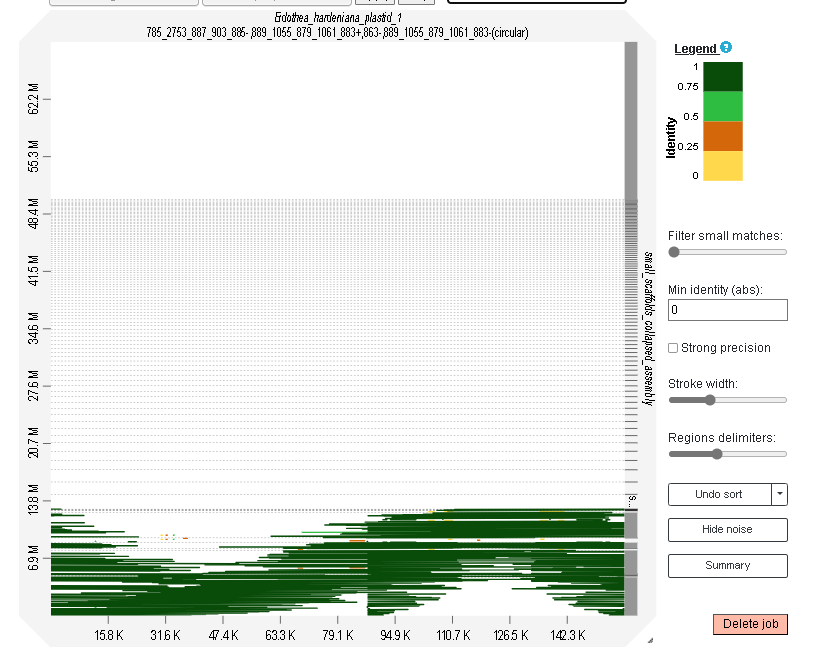


**Figure S25:** Small unplaced scaffolds aligned against the chloroplast genome.


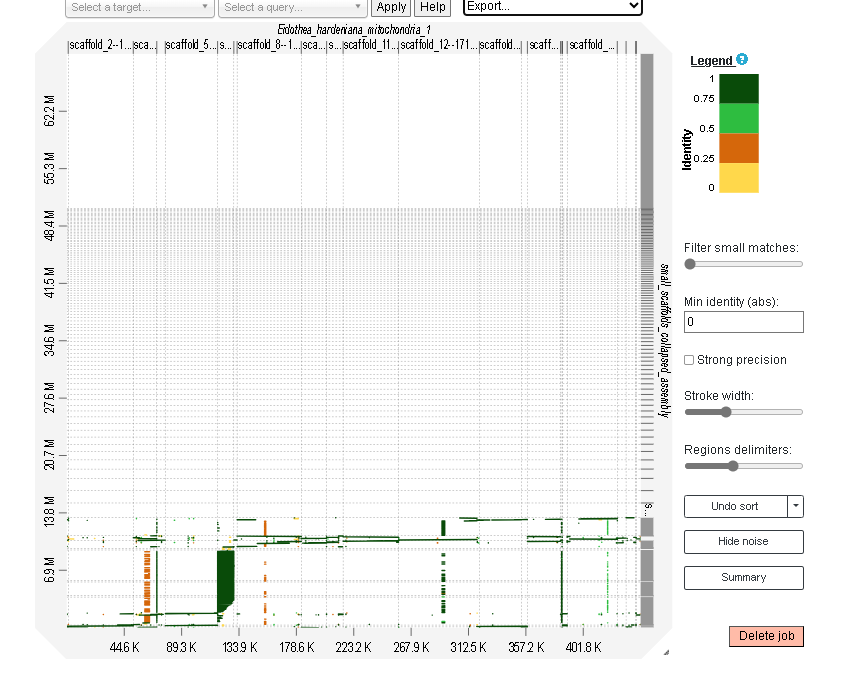


**Figure S26:** Small unplaced scaffolds aligned against the mitochondrial genome.


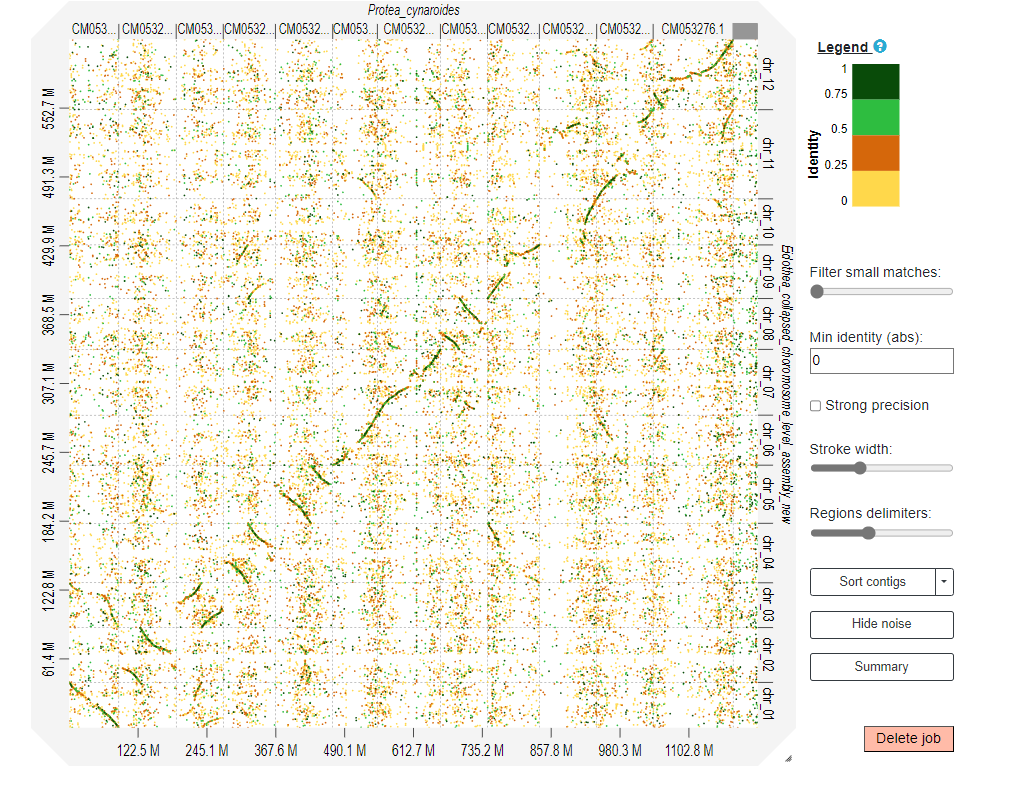


**Figure S27:** Consensus genome assembly of *Eidothea hardeniana* against the reference genome *Protea cynaroides*, another member of Proteoideae subfamily of the Proteaceae family


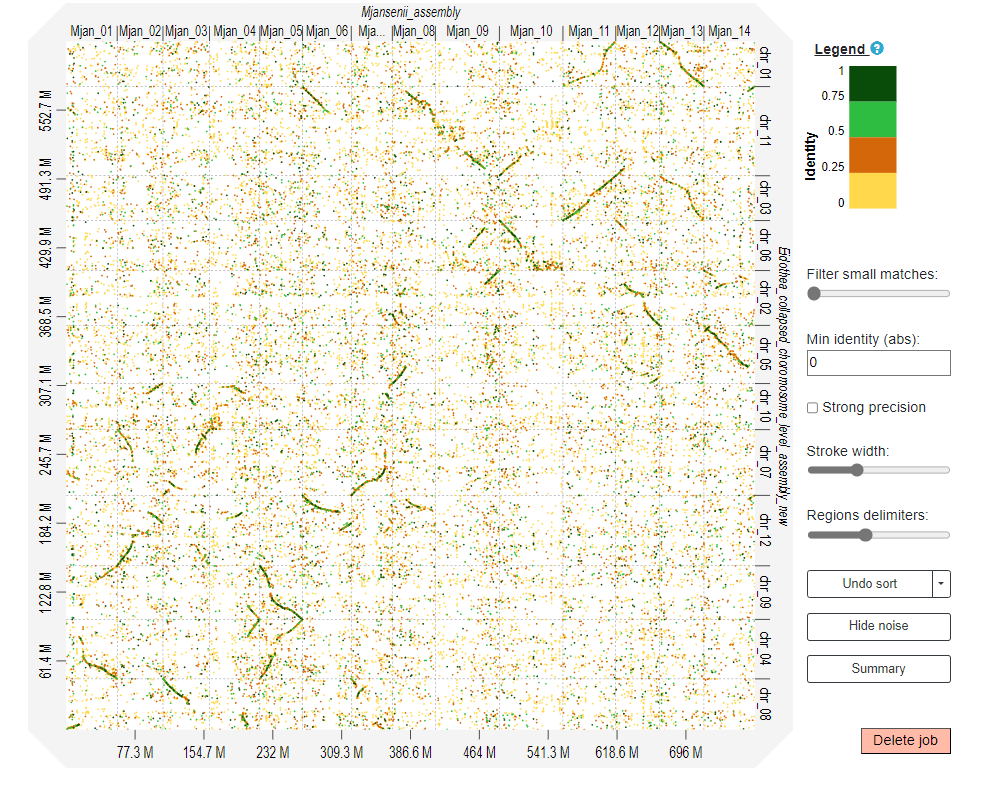


**Figure S28**: Consensus genome assembly of the *Eidothea hardeniana* against *Macadamia jansenii*, member of Grevilleoideae subfamily of the Proteaceae family.


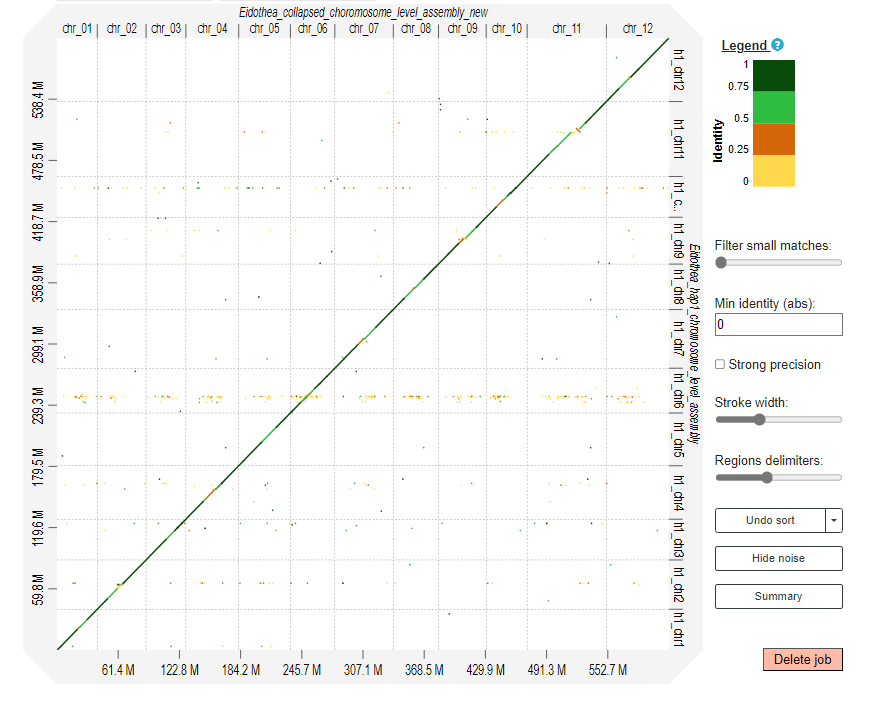


**Figure S29**: Dot‐plot comparison of two Eidothea chromosome‐level genome assemblies. The x‐axis (bottom) shows the consensus assembly, labelled by chromosome (chr_01 through chr_12) with approximate lengths in megabases (Mb), and the y‐axis (left) shows the haplotype 1 assembly. Each point in the plot corresponds to an alignment between the two assemblies; points along the main diagonal indicate syntenic regions (i.e., regions that align in the same order and orientation). The color scale (right) represents sequence identity, ranging from yellow (lower identity) through orange to green (higher identity). Off‐diagonal points typically represent rearrangements, duplications or other structural differences between the assemblies.


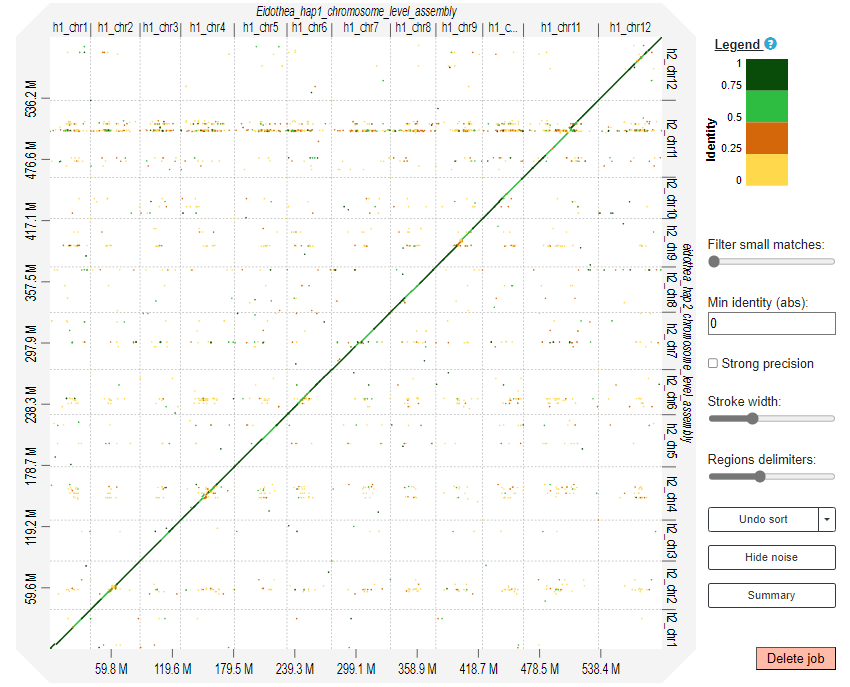


**Figure S30** Dot‐plot comparison of two Eidothea chromosome‐level genome assemblies. The x‐axis (bottom) shows the Haplotype 1 assembly, labelled by chromosome (chr_01 through chr_12) with approximate lengths in megabases (Mb), and the y‐axis (left) shows the haplotype 2 assembly. Each point in the plot corresponds to an alignment between the two assemblies; points along the main diagonal indicate syntenic regions (i.e., regions that align in the same order and orientation). The color scale (right) represents sequence identity, ranging from yellow (lower identity) through orange to green (higher identity). Off‐diagonal points typically represent rearrangements, duplications or other structural differences between the assemblies.


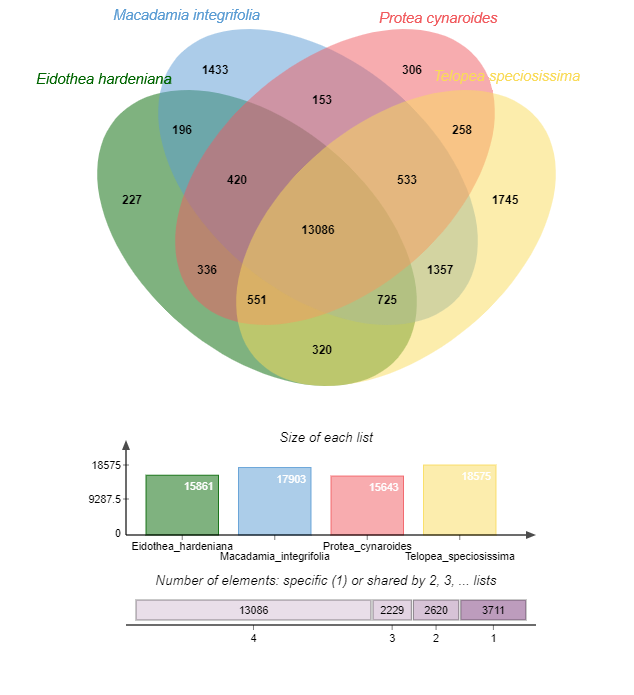


**Figure S31** Comparative orthogroup analysis across four Proteaceae species. Venn Diagram showing shared and unique orthologous gene clusters among *Eidothea hardeniana, Macadamia integrifolia, Protea cynaroides, and Telopea speciosissima.*


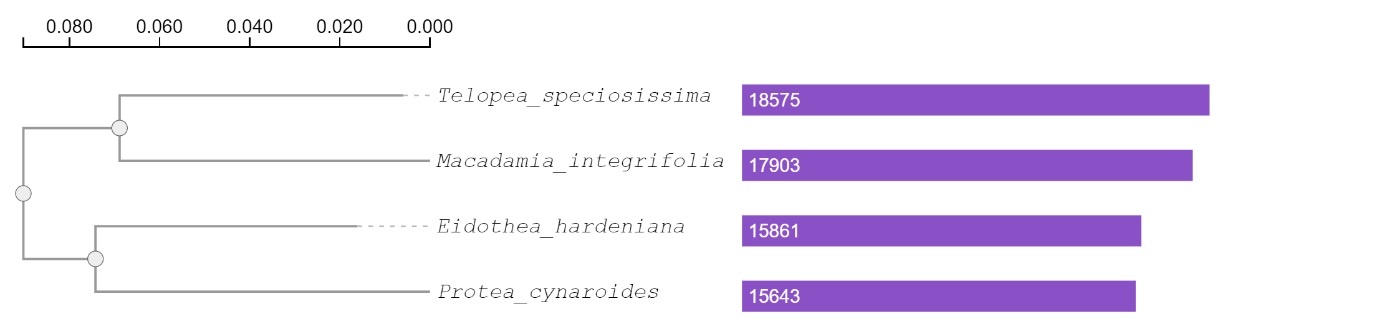
 **Figure S32** Phylogenetic relationships and total orthogroup counts among four Proteaceae species. Species tree depicting the evolutionary relationships between *Telopea speciosissima*, *Macadamia integrifolia*, *Eidothea hardeniana*, and *Protea cynaroides*, inferred from shared orthogroups. Bar plots indicate the total number of orthologous gene clusters identified per species. *Eidothea hardeniana* retains 15,861 orthogroups, comparable to its close relatives, suggesting no major gene loss despite its demographic isolation and low heterozygosity.


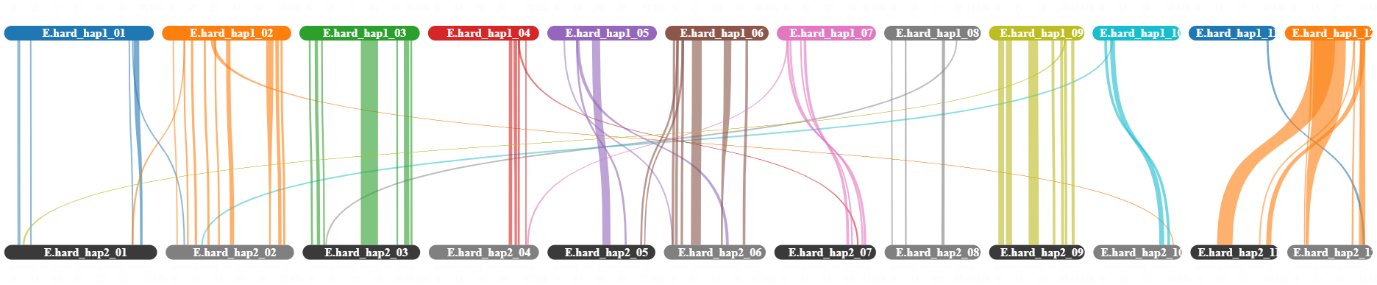


**Figure S33** Syntenic relationships between chromosome-level assemblies of haplotype 1 genome (top) and Haplotype 2 (bottom) of *Eidothea hardeniana*, visualized using MCScanX. Each ribbon represents a collinear block of orthologous genes between corresponding or non-corresponding chromosomes. The syntenic links indicate strong collinearity across genomes. Inter-chromosomal connections and sparse may suggest minor rearrangements, assembly artifacts, or collapsed repetitive regions.


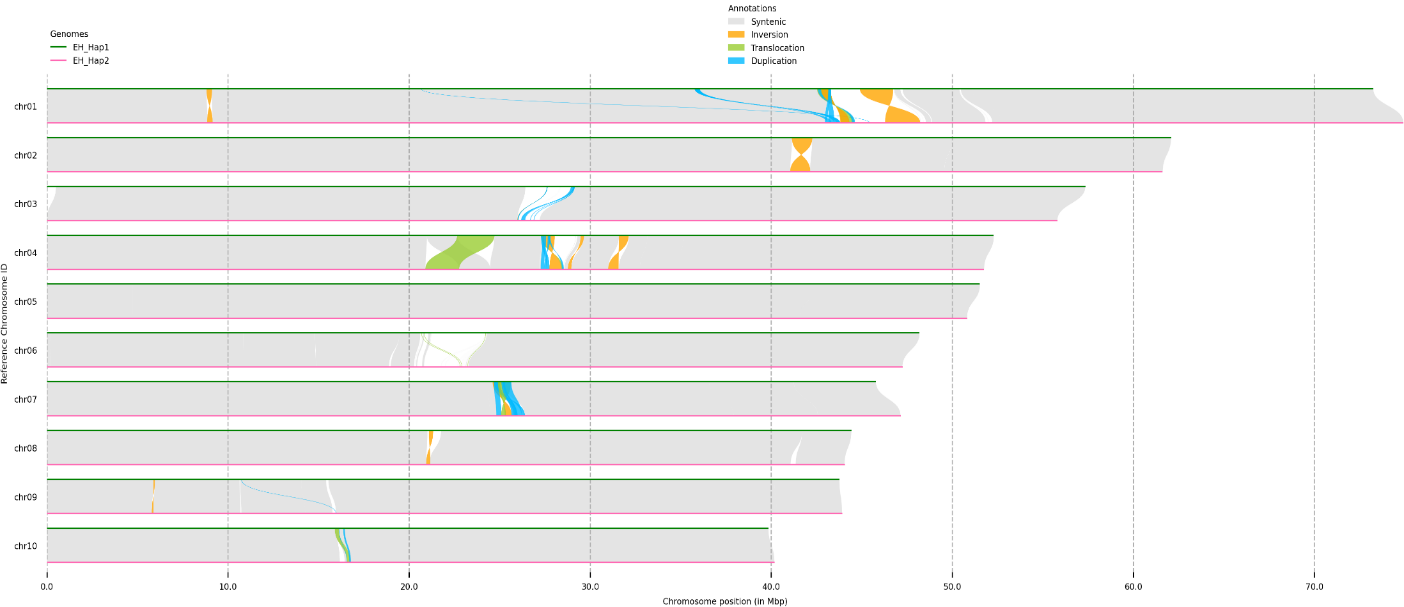


**Figure S34** Structural comparison between haplotyp1 (orange track) and haplotype 2 (green track) using SyRI. Syntenic regions (gray) dominate the alignment, indicating strong collinearity between the two genomes.


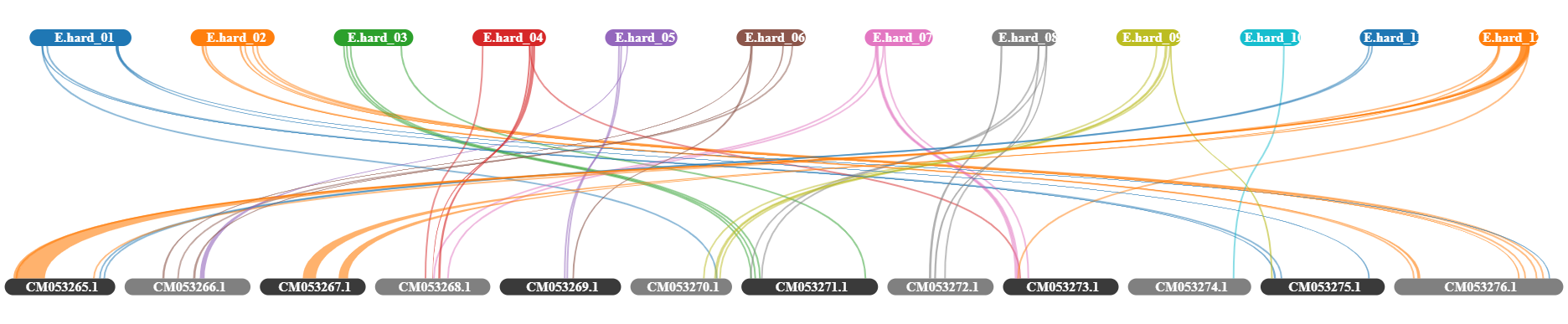


**Figure S35** Syntenic relationships between the chromosome-level assemblies of Eidothea hardeniana (top) and Protea cynaroides (bottom) as identified by MCScanX. Colored links represent conserved collinear blocks across the two genomes. Despite significant divergence time, several chromosomes (e.g., E.hard_01 with CM053265.1, E.hard_02 with CM053266.1) show conserved syntenic blocks, suggesting chromosomal stability across deep evolutionary timescales. However, extensive interchromosomal rearrangements and translocations are also evident, reflecting lineage-specific genomic reorganization.


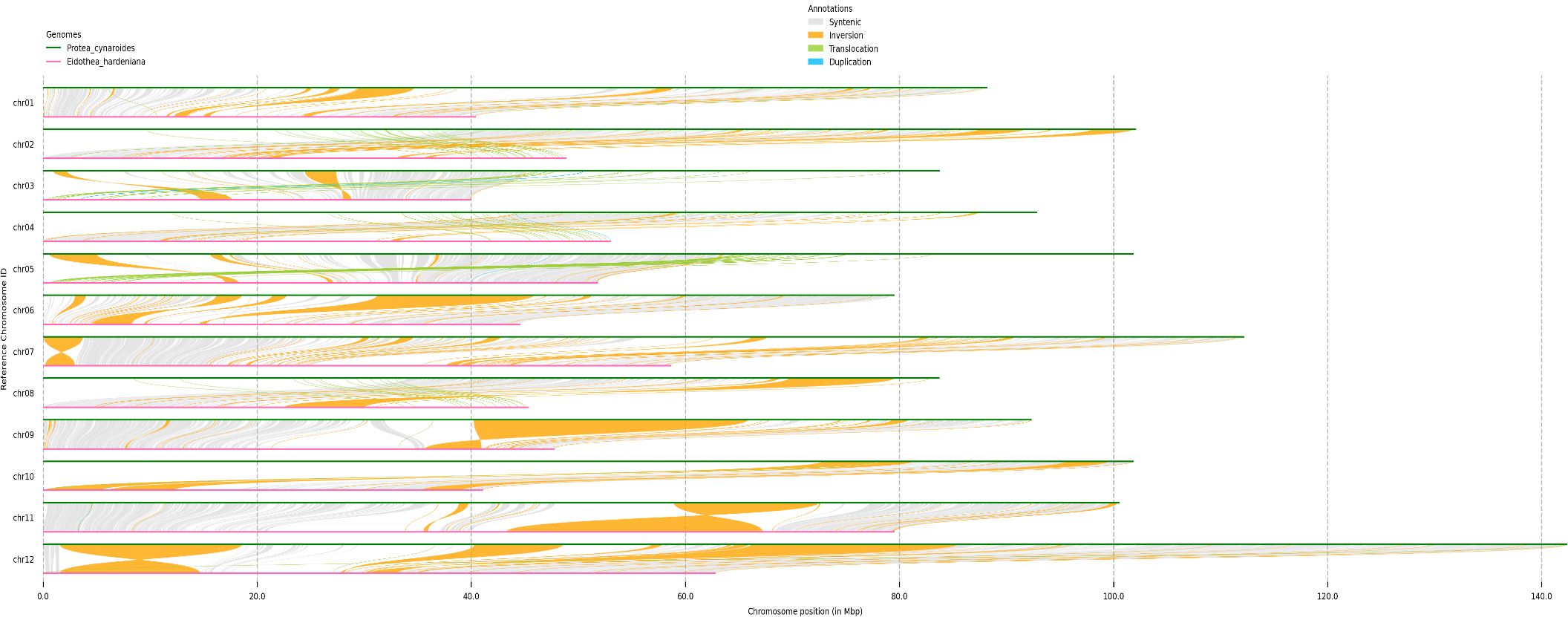
 **Figure S36** Genome-wide structural comparison between *Eidothea hardeniana* (pink) and *Protea cynaroides* (green) using SyRI. The reference genome is *Protea cynaroides* (x-axis), and the aligned query is *Eidothea hardeniana* (y-axis). Colored blocks represent different structural features: syntenic regions (gray), inversions (orange), translocations (green), and duplications (blue). The results reveal a mixture of conserved collinearity interspersed with large-scale inversions and several translocations. These rearrangements reflect substantial structural divergence between the two lineages since their evolutionary separation, despite belonging to the same subfamily (Proteoideae). Chromosomes such as chr03, chr05, and chr07 exhibit particularly high levels of rearrangement, highlighting lineage-specific genome evolution in *Eidothea*.


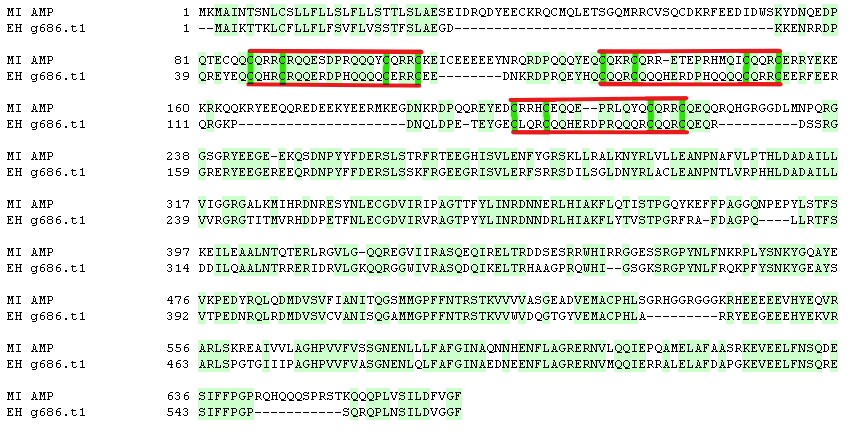


**Figure S37** Blast analysis confirming the functional conservation of the MiAMP2 gene in E. hardeniana

**Figure S38** Effect of variant frequency threshold on total number of SNVs detected in the E. hardeniana genome. The plot shows the number of detected SNV positions (including both heterozygous and homozygous variants) across different minimum frequency thresholds (x-axis).
