# Supplementary material for: Haplotype-Resolved Genome of the Critically Endangered, Paleo-endemic Tree, Eidothea hardeniana
Source: Genome Biol Evol. 2026 Mar 19;18(4):evag071. doi: 10.1093/gbe/evag071 (PMC13080361; doi:10.1093/gbe/evag071)
Supplement: evag071_Supplementary_Data [file evag071_supplementary_data.zip › Supplementary_Methods_S1_without_track_changes.docx]

**Haplotype-resolved genome of the critically endangered, paleo-endemic tree, *Eidothea hardeniana***

Authors: Abhishek Soni^1,2^, Agnelo Furtado^1,2^, Maurizio Rossetto^3,4^, Robert J. Henry^1,5*^

[^a.soni@uq.edu.au (A.S.)^](mailto:a.soni@uq.edu.au%20(A.S.))^,^ [^a.furtado@uq.edu.au^](mailto:a.furdato@uq.edu.au) ^(A.F.),^ [^maurizio.rossetto@botanicgardens.nsw.gov.au^](mailto:maurizio.rossetto@botanicgardens.nsw.gov.au) ^(M.R.),^ [^robert.henry@uq.edu.au^](mailto:robert.henry@uq.edu.au) ^(R.J.H.)^

1. ARC Centre of Excellence for Plant Success in Nature and Agriculture, The University of Queensland, St Lucia 4072 QLD Australia
2. Centre for Crop Science, The Queensland Alliance for Agriculture and Food Science, The University of Queensland, St Lucia 4072 QLD Australia
3. Research Centre for Ecosystem Resilience, Royal Botanic Garden Sydney, Sydney 2000 NSW Australia
4. The University of Queensland, St Lucia 4072 QLD Australia
5. VinUni Big Data Research Institute VinUniversity, Hanoi, Vietnam

*Correspondence: Prof Robert Henry (R.J.H.) [robert.henry@uq.edu.au](mailto:robert.henry@uq.edu.au)

**Supplementary Methods S1**

**Sample collection**

Young green leaves of Eidothea hardeniana (accession no. AA 20010147) were collected from the Royal Botanic Garden Sydney, Australia. For flow cytometry-based genome size estimation, approximately 10 g of fresh leaf tissue was wrapped in moist paper towels, placed in a plastic container to prevent desiccation and mechanical damage, and transported overnight to the University of Queensland, Brisbane, Australia. For DNA and RNA extractions, approximately 50 g of leaf tissue was flash-frozen in liquid nitrogen, stored at –80 °C without thawing, and transported under cold conditions to the University of Queensland. An additional about 20 g of frozen tissue was shipped on dry ice to Arima Genomics, The Australian National University, Canberra, Australia for Hi-C library preparation.

**Genome size and ploidy estimation**

Both Flow cytometry (FCM) and k-mer based approaches were used to estimate the genome size of the *E. hardeniana*. For FCM, 40mg of fresh leaf material of *E. hardeniana* and 20 mg of Nipponbare rice was co-chopped as previously described(Doležel, et al. 2007; Soni and Henry 2024). The chopping buffer (MgSO_4_ buffer) was modified with 3% PVP-10 to avoid the impact of secondary metabolites(Jordan, et al. 2015). The sample fluorescence was measured on a Becton Dickinson Fortessa X20 Cell Analyzer (BD Biosciences, San Jose, CA, USA) as per(Soni, et al. 2025). The 1C value of the internal standard was determined by averaging the genome size of two haplotypes(Soni and Henry 2024). For k-mer-based genome size estimation, Kmergenie v1.7051 (Chikhi and Medvedev 2014) was used to identify the optimal k-mer size and obtain an initial genome size estimate, whereas GenomeScope 2.0 (Ranallo-Benavidez, et al. 2020) was used to estimate genome size and heterozygosity from the k-mer frequency distribution. To infer the ploidy level of *E. hardeniana*, k-mer-based analyses were performed by counting k-mers with Jellyfish(Marçais and Kingsford 2011), and the resulting k-mer histograms were subsequently analysed with Smudgeplot (v 0.2.5) (Ranallo-Benavidez, et al. 2020)to distinguish among alternative ploidy scenarios (e.g., diploid, polyploid).

**DNA extraction, sequencing and quality control**

For HiFi and ONT sequencing, leaf material was finely pulverized with Qiagen TissueLyser II (Qiagen, Hilden, Germany) by avoiding thawing. Subsequently, high molecular weight DNA was extracted by a modified CTAB method available as GIH_SOP204-02 (https://dx.doi.org/10.17504/protocols.io.b5qyq5xw). The integrity and purity of extracted DNA were assessed using agarose gel electrophoresis, spectrophotometer and Qubit. Tapestation 4150 (Agilent Technologies, Santa Clara, CA, USA) was used to confirm the intactness of the DNA. Subsequently, 20 µg HMW DNA was sheared to 15 kb using the Megaruptor 3 system (Diagenode, Denville, NJ, USA). Libraries were prepared using the PacBio SMRTbell Express Template Prep Kit 2.0 (Pacific Biosciences, Menlo Park, CA, USA) following the manufacturer’s instructions. Sequencing was performed on the PacBio Sequel II platform at Genome Innovation Hub at the University of Queensland. PacBio HiFi sequencing produced ~42× coverage (mean read length 12.7 kb, N50 = 14.8 kb) (Figure S1).

In addition, 10 µg HMW DNA was prepared for ONT sequencing using the Ligation Sequencing Kit (SQK-LSK114, Oxford Nanopore Technologies, Oxford, UK). Before library preparation, size selection was performed using a Circulomics short read eliminator kit (Circulomics, Baltimore, MD, USA). Libraries were loaded onto R10.4.1 PromethION flow cells on PromethION 2 (P2solo) device and base called with Guppy 657, performed with "dna_r10.4.1_e8.2_400bps_sup.cfg" configuration. The library was run until the pores died. ONT sequencing provided ~167× coverage (mean read length 18.7 kb, N50 = 27.6kb) with ultra-long reads up to 3.5 Mb (Figure S2). The quality of the HiFi and ONT reads was assessed with fastQC (v. 0.12.1) (https://github.com/s-andrews/FastQC). HiFi reads were processed for adapter filter contamination with HifiAdapterFilt (v. 3.0.0) (https://github.com/sheinasim-USDA/HiFiAdapterFilt).

Hi-C library was generated using the high coverage Hi-C kit (Arima Genomics, San Diego, CA, USA) following the manufacturer’s standard protocol. Briefly, freshly isolated nuclei were crosslinked to preserve native chromatin architecture and then digested with a proprietary enzyme cocktail which cuts at ^GATC, G^ANTC, C^TNAG, and T^TAA sites. The crosslinked DNA fragments were proximity-ligated, capturing genomic regions that were spatially close within the nucleus. After reversing crosslinks, the resulting library underwent size-selection and purification steps before being amplified. The Hi-C library was then sequenced on an Illumina NovaSeq 6000 platform (Illumina, San Diego, CA, USA) with 150 bp paired-end reads, yielding long-range interaction data critical for scaffolding and resolving complex regions within the genome. A total of 115Gb of Hi-C data (768 million reads) was generated. Hi-C reads were quality and length trimmed using fastp (v. 0.23.4)(Chen 2023; Chen, et al. 2018). Reads shorter than 75 bp, reads with average Phred quality score (< Q30), and reads containing more than one ambiguous base (N) were discarded. In addition, the first five bases at the 5′ end of both forward and reverse reads were removed in accordance with the Arima Genomics mapping guidelines (Doc A160156 v03; <https://github.com/ArimaGenomics/mapping_pipeline>). After trimming, 100Gb of Hi-C data (712 million read pairs) were retained for downstream analysis.

In addition to the HiFi, ONT, and Hi-C datasets, short-read whole-genome sequencing was performed using the DNBseq platform (BGI Genomics, Shenzhen, China). Approximately 2 µg of high-quality genomic DNA was used to prepare a PCR-free library following BGI’s standard protocol. Circular DNA nanoballs (DNBs) were generated and sequenced using combinatorial Probe-Anchor Synthesis (cPAS) chemistry. Base calling and quality filtering were performed using BGI’s in-house pipeline. Subsequently, the reads were quality and length trimmed using fastp (v. 0.23.4). Reads shorter than 75 bp, reads with average Phred quality score (< Q30), and reads containing more than one ambiguous base (N) were discarded. In addition, short reads were subjected to Kraken2 classification (Lu, et al. 2022; Wood, et al. 2019) against the NCBI database and reads assigned to non-plant taxa were removed prior to downstream analyses. The resulting paired-end reads were used for k-mer-based genome size estimation, ploidy inference, and variant analysis.

**RNA extraction and sequencing**

RNA was extracted from frozen leaf material using a modified RNA extraction protocol(Rubio-Piña and Zapata-Pérez 2011; Wang and Stegemann 2010). CTAB buffer was prepared (Rubio-Piña and Zapata-Pérez 2011) and RNA was extracted(Wang and Stegemann 2010). RNA was purified using Qiagen's RNeasy Mini kit(Furtado 2014). In brief, 500 mg of frozen leaf material was lysed in the prewarmed (65°C) CTAB buffer topped with 10% beta-mercaptoethanol. Extracted RNA was treated with DNase and filtered using Qiagen Plant Mini RNase kit (Qiagen, Hilden, Germany). The quality of the RNA was determined using a NanoDrop spectrophotometer (Thermo Fisher Scientific, Waltham, MA, USA) with 260/280 ratio of 2-2.2 and 260/230 ratio of 1.8-2.0. Further the integrity of the RNA was analysed using an Agilent Bioanalyzer (Agilent Technologies, Santa Clara, CA, USA). RNA was sequenced on an Illumina NovaSeq platform (Illumina, San Diego, CA, USA) at the Australian Genomic Research Facility, University of Queensland, Brisbane, Australia. Base calling was performed using NovaSeq Control Software v.1.1.0 and RTA v.4.6.2, and FASTQ files were generated via DRAGEN BCL convert pipeline v.4.0.3. A total of 420 million reads (63Gb) were obtained. Reads were trimmed using fastp (v. 0.23.4) (Chen 2023; Chen, et al. 2018), removing those with average quality <Q30, >1 ambiguous base (N), length <75 bp, or >40% unqualified bases. After QC, 357 million reads were retained for downstream analysis.

**The role of ONT read length in assembly quality**

Ultra-long ONT reads (>100 kb) have been critical for assembling highly contiguous genomes(Koren, et al. 2024; Lu, et al. 2024; Nurk, et al. 2022; Shang, et al. 2023), yet repetitive regions such as rDNA arrays, telomeres, and AT-rich regions often remain unresolved when ONT is used alone(Koren, et al. 2024). Hybrid strategies that integrate ONT, PacBio HiFi, and Hi-C data leverage the strengths of each technology: HiFi provides base accuracy, Hi-C delivers chromosome-scale scaffolding, and ONT spans complex repeats(Espinosa, et al. 2024; Lu, et al. 2024; Nurk, et al. 2022; Shang, et al. 2023). Here, extra-long ONT reads with 22× coverage were used to resolve complex regions. Generating long ONT reads remains challenging in recalcitrant species due to DNA degradation and secondary metabolites, which limits long read recovery(Espinosa, et al. 2024).

**Genome assembly, scaffolding and manual curation**

Several high-quality genomes have been assembled using PacBio HiFi, Hi-C and ultra- long ONT reads(Chen, et al. 2023; Koren, et al. 2024; Nurk, et al. 2022; Shang, et al. 2023). In many non-model species, mechanical and enzymatic limitations prevent the recovery of ultra-long ONT reads (>100 kb)(Espinosa, et al. 2024; Koren, et al. 2024). As an alternative, extra-long ONT reads (>50 kb) were used in this study.

Different approaches were used to assemble the genome: using only HiFi + Hi-C reads, using ONT + Hi-C reads and using ONT, HiFi and Hi-C reads integration. Initially, a hybrid assembly of HiFi and Hi-C reads was generated using HiFiasm (v 0.19.8) (Cheng, et al. 2021)with default parameters. Subsequently, another assembly was created using the similar dataset but also integrating the complete ONT dataset (ONT_unfiltered). Additional assemblies were generated by selectively including ONT reads above various length thresholds (>20 kb, >30 kb, >40 kb, >50 kb), filtered using seqkit (v2.7.0)(Shen, et al. 2016). Before scaffolding, each assembly was assessed for BUSCO completeness, N50 and number of chromosome-length contigs.

The contig-level assemblies were subjected to the scaffolding tool YaHS (v 1.2.1 )(Zhou, et al. 2023). Before scaffolding, Hi-C reads were aligned to the reference genome assembly generated by HiFiasm using bwa (v 0.7.17)(Li 2013). Mapping was performed separately for R1 and R2 reads and the 5′ ends of chimeric Hi-C reads were filtered using the filter_five_end.pl script (<https://github.com/ArimaGenomics/mapping_pipeline.git>). The resulting single-end reads were then paired with two_read_bam_combiner.pl (<https://github.com/ArimaGenomics/mapping_pipeline.git>), generating a sorted, mapping-quality–filtered paired-end BAM file for each sequencing lane. Finally, read groups were assigned to the BAM files using Picard toolkit (https://broadinstitute.github.io/picard). The contig error correction module in YaHS was not used, in order to maintain the base-level accuracy of the HiFi reads assembled and corrected with haplotype-resolved error correction algorithm (Cheng, et al. 2021). Following assembly, the assembly was screened for potential contamination. Taxonomic classification of scaffolds was performed using Kraken2 (Lu, et al. 2022; Wood, et al. 2019) against the NCBI nucleotide database. Given that the final assembly consisted of chromosome-length scaffolds, particular attention was given to any scaffolds assigned to non-plant taxa.

Subsequently, the consensus genome assembly of *Eidothea hardeniana* was aligned against *Protea cynaroides* reference genome (a South African member of the Proteoideae subfamily(Chang, et al. 2023)) using minimap2 (v2.28)(Li 2018). Dot plots were generated using D-GENIES (Cabanettes and Klopp 2018) to validate scaffolding. Highly collinear scaffolds were joined in CLC Genomics Workbench v24, with 100 Ns inserted at junctions, following the standard gap-padding procedures used by YaHS. Scaffolds in which telomeric signals appeared internally were reverse-complemented and re-joined to ensure correct orientation.

The quality of the genome assembly was evaluated by assessing BUSCO gene completeness against the embryophyta_odb10 database using BUSCO (v5.4.7)(Seppey, et al. 2019; Simão, et al. 2015), and assembly metrics were calculated by QUAST v5.0.2(Gurevich, et al. 2013). Chromosome-scale structure was further evaluated using the QuarTeT toolkit v1.2.5. Telomeric motifs (‘TTTAGGG’) were profiled along each scaffold with TeloExplorer(Brown, et al. 2023), and candidate centromeric regions were inferred with CentroMiner. For the YaHS-scaffolded assembly, .hic files were produced with Juicer (Durand, et al. 2016b) and visualised in Juicebox v2.17(Durand, et al. 2016a). For the final curated assembly, Hi-C reads were remapped to the consensus genome and contact maps were generated with HiCExplorer v3.7.6(Wolff, et al. 2022). High-resolution per-scaffold contact maps were additionally created with PretextMap (v.0.1.9) (https://github.com/sanger-tol/PretextMap) and inspected in PretextView v1.0.5 to verify scaffold continuity.

**Repetitive element evaluation**

For repeat elements, Repeatmodeler2 (v. 2.0.1) (Flynn, et al. 2020)was used to identify the repetitive elements de novo and “soft-masked” masked with repeat masker (v. 4.1.7)(Chen 2004).

**Gene prediction and functional annotation**

Protein-coding genes were predicted using the BRAKER3 pipeline (ab initio mode)(Gabriel, et al. 2024). RNA-seq reads were first aligned to the repeat-masked genome using HISAT2 and provided to BRAKER3 as evidence for gene prediction. Additional peptide evidence from *Viridiplantae* was also incorporated to improve model accuracy. Protein homology evidence was derived from the Viridiplantae odb v11 protein dataset obtained from OrthoDB v11 using orthodb-clades (<https://github.com/tomasbruna/orthodb-clades>). The dataset included representative angiosperm species spanning major eudicot lineages, including available Proteaceae proteomes where publicly available. Predicted protein sequences were assessed for BUSCO completeness following retention of only the longest isoform per gene locus. The predicted coding sequences (CDSs) were functionally annotated using OmicsBox v2.2.4 (<https://www.biobam.com/omicsbox>. Functional assignment was performed in **BLASTx-fast mode** against the **NCBI non-redundant protein database (nr v5),** applying an e-value threshold of 1e-10, a maximum of 10 hits per query, and a taxonomy filter restricted to Viridiplantae (taxon ID: 33090). For sequences with significant BLAST hits, **Gene Ontology (GO) terms** and **InterProScan domains** were retrieved and annotated. GO terms obtained from Blast2GO mapping (Conesa, et al. 2005) were merged with those derived from InterProScan (Jones, et al. 2014) to produce integrated functional annotations. CDSs without BLAST matches were further assessed for coding potential using the **Coding Potential Assessment Tool (CPAT)** implemented in OmicsBox. Models were trained on Arabidopsis thaliana along with a M. *integrifolia* model to refine classification of protein-coding versus non-coding transcripts.

**Gene family identification**

Sequences annotated in OmicsBox were further screened for specific functional categories, including antimicrobial peptides and flowering-related genes. Candidate genes were identified through BLAST-based homology searches. Homologs of antimicrobial peptides were then compared with those of *Macadamia integrifolia* by sequence alignment using Clone Manager 9(Sharma, et al. 2024).

**Variant calling and heterozygosity**

For the assessment of the genetic diversity within the species, the BGI short read sequencing data was generated from the same plant from which the genome assembly was generated. The trimmed short read data corresponded to ~31x coverage of the *E. hardeniana* genome (assembled genome size = 614 Mbp). The variant calling was accomplished with Qiagen CLC Genomics Workbench v. 24 (CLC bio, Aarhus, Denmark), where the trimmed reads were first mapped to the reference genome with a range of combinations of length fraction (1, 0.95, 0.9, 0.8) and similarity fraction (1, 0.95, 0.9, 0.8). Other parameters, match score (1), mismatch score (2), insertion and deletion cost (3) were kept consistent for the different runs of mapping. Because allelic balance can vary depending on sequencing depth, particularly at lower coverage levels where heterozygous sites may deviate from the expected 50:50 ratio, multiple variant allele frequency thresholds (15–40%) were evaluated to ensure conservative and robust SNP detection. After refining for 100% homozygous and high-confidence heterozygous calls, 719,174 SNV positions were retained. Homozygous variant positions were defined as sites where all mapped reads supported the alternate allele relative to the reference genome.

**Orthologous gene analysis**

To identify shared and lineage-specific gene clusters, orthologous clustering was performed using Orthovenn3 (https://orthovenn3.bioinfotoolkits.net/) with OrthoFinder algorithm (v2.5.4) (Emms and Kelly 2019)on predicted protein sequences from *Eidothea hardeniana*, *Protea cynaroides* (GCA_028583415.1), *Macadamia integrifolia(Sharma, et al. 2024)*, and *Telopea speciosissima* (GCA_018873765.1). Protein sets were filtered to retain the longest isoform per gene prior to analysis.

**Synteny and collinearity analysis**

To identify collinear blocks and syntenic relationships, protein sequences from each assembly were first reduced to the longest isoform per gene and searched against each other using Diamond (v. 2.1.13) Blastp (Buchfink, et al. 2021) with default parameters optimized for speed and sensitivity. The resulting BLAST output was renamed with the prefix of the target species. Using blast files and BRAKER3-predicted GFF3 annotations files, collinearity files were generated using MCScanX (v. 1.0) (Wang, et al. 2012)and visualized using SynVisio(Bandi and Gutwin 2020).

**Chromosomal rearrangement analysis**

Structural rearrangements, including inversions, translocations, and duplications, were identified using SyRI (v1.6)(Goel, et al. 2019). Pairwise whole-genome alignments were first generated with minimap2 (v. 2.28)(Li 2018), and the resulting alignment files were sorted and indexed with samtools (Li, et al. 2009) to produce BAM input for SyRI. The SyRI outputs were then parsed to classify structural variation types, and candidate rearrangements were validated using dot plots.

**References**

Bandi V, Gutwin C editors. Graphics Interface 2020. 2020.

A Telomere Identification Toolkit [Internet]. Zenodo2023 [cited 2024 02/10/2024]. Available from: <https://zenodo.org/records/10091385>

Buchfink B, Reuter K, Drost H-G 2021. Sensitive protein alignments at tree-of-life scale using DIAMOND. Nature Methods 18: 366-368. doi: 10.1038/s41592-021-01101-x

Cabanettes F, Klopp C 2018. D-GENIES: dot plot large genomes in an interactive, efficient and simple way. PeerJ 6: e4958.

Chang J, et al. 2023. The genome of the king protea, *Protea cynaroides*. PLANT JOURNAL 113: 262-276. doi: 10.1111/tpj.16044

Chen J, et al. 2023. A complete telomere-to-telomere assembly of the maize genome. Nature genetics 55: 1221-1231. doi: 10.1038/s41588-023-01419-6

Chen N 2004. Using Repeat Masker to identify repetitive elements in genomic sequences. Current protocols in bioinformatics 5: 4.10. 11-14.10. 14.

Chen S 2023. Ultrafast one‐pass FASTQ data preprocessing, quality control, and deduplication using fastp. Imeta 2: e107.

Chen S, Zhou Y, Chen Y, Gu J 2018. fastp: an ultra-fast all-in-one FASTQ preprocessor. Bioinformatics 34: i884-i890.

Cheng H, Concepcion GT, Feng X, Zhang H, Li H 2021. Haplotype-resolved de novo assembly using phased assembly graphs with hifiasm. Nature Methods 18: 170-175.

Chikhi R, Medvedev P 2014. Informed and automated k-mer size selection for genome assembly. Bioinformatics 30: 31-37.

Conesa A, et al. 2005. Blast2GO: a universal tool for annotation, visualization and analysis in functional genomics research. Bioinformatics 21: 3674-3676.

Doležel J, Greilhuber J, Suda J 2007. Estimation of nuclear DNA content in plants using flow cytometry. Nature Protocols 2: 2233-2244. doi: 10.1038/nprot.2007.310

Durand NC, et al. 2016a. Juicebox provides a visualization system for Hi-C contact maps with unlimited zoom. Cell systems 3: 99-101.

Durand NC, et al. 2016b. Juicer provides a one-click system for analyzing loop-resolution Hi-C experiments. Cell systems 3: 95-98.

Emms DM, Kelly S 2019. OrthoFinder: phylogenetic orthology inference for comparative genomics. Genome Biology 20: 238.

Espinosa E, Bautista R, Larrosa R, Plata O 2024. Advancements in long-read genome sequencing technologies and algorithms. Genomics 116. doi: 10.1016/j.ygeno.2024.110842

Flynn JM, et al. 2020. RepeatModeler2 for automated genomic discovery of transposable element families. Proceedings of the National Academy of Sciences 117: 9451-9457.

Furtado A 2014. RNA extraction from developing or mature wheat seeds. Cereal genomics: Methods and protocols: 23-28.

Gabriel L, et al. 2024. BRAKER3: Fully automated genome annotation using RNA-seq and protein evidence with GeneMark-ETP, AUGUSTUS, and TSEBRA. Genome research 34: 769-777.

Goel M, Sun H, Jiao W-B, Schneeberger K 2019. SyRI: finding genomic rearrangements and local sequence differences from whole-genome assemblies. Genome Biology 20: 277.

Gurevich A, Saveliev V, Vyahhi N, Tesler G 2013. QUAST: quality assessment tool for genome assemblies. Bioinformatics 29: 1072-1075.

Jones P, et al. 2014. InterProScan 5: genome-scale protein function classification. Bioinformatics 30: 1236-1240.

Jordan GJ, Carpenter RJ, Koutoulis A, Price A, Brodribb TJ 2015. Environmental adaptation in stomatal size independent of the effects of genome size. New Phytologist 205: 608-617. doi: 10.1111/nph.13076

Koren S, et al. 2024. Gapless assembly of complete human and plant chromosomes using only nanopore sequencing. Genome research 34: 1919-1930.

Li H 2013. Aligning sequence reads, clone sequences and assembly contigs with BWA-MEM. arXiv preprint arXiv:1303.3997.

Li H 2018. Minimap2: pairwise alignment for nucleotide sequences. Bioinformatics 34: 3094-3100.

Li H, et al. 2009. The sequence alignment/map format and SAMtools. Bioinformatics 25: 2078-2079.

Lu D, et al. 2024. Nanopore ultra-long sequencing and adaptive sampling spur plant complete telomere-to-telomere genome assembly. Molecular Plant 17: 1773-1786. doi: 10.1016/j.molp.2024.10.008

Lu J, et al. 2022. Metagenome analysis using the Kraken software suite. Nature Protocols 17: 2815-2839.

Marçais G, Kingsford C 2011. A fast, lock-free approach for efficient parallel counting of occurrences of k-mers. Bioinformatics 27: 764-770. doi: 10.1093/bioinformatics/btr011

Nurk S, et al. 2022. The complete sequence of a human genome. Science 376: 44-53. doi: 10.1126/science.abj6987

Ranallo-Benavidez TR, Jaron KS, Schatz MC 2020. GenomeScope 2.0 and Smudgeplot for reference-free profiling of polyploid genomes. Nature Communications 11: 1432.

Rubio-Piña JA, Zapata-Pérez O 2011. Isolation of total RNA from tissues rich in polyphenols and polysaccharides of mangrove plants. Electronic journal of Biotechnology 14: 11-11.

Seppey M, Manni M, Zdobnov EM 2019. BUSCO: assessing genome assembly and annotation completeness. Gene prediction: methods and protocols: 227-245.

Shang L, et al. 2023. A complete assembly of the rice Nipponbare reference genome. Molecular Plant 16: 1232-1236. doi: 10.1016/j.molp.2023.08.003

Sharma P, et al. 2024. Genome sequences to support conservation and breeding of Macadamia. Tropical Plants 3. doi: 10.48130/tp-0024-0029

Shen W, Le S, Li Y, Hu F 2016. SeqKit: a cross-platform and ultrafast toolkit for FASTA/Q file manipulation. PLOS ONE 11: e0163962.

Simão FA, Waterhouse RM, Ioannidis P, Kriventseva EV, Zdobnov EM 2015. BUSCO: assessing genome assembly and annotation completeness with single-copy orthologs. Bioinformatics 31: 3210-3212.

Soni A, Constantin L, Furtado A, Henry RJ 2025. A Flow Cytometry Protocol for Measurement of Plant Genome Size Using Frozen Material. Applied Biosciences 4: 28. doi: 10.3390/applbiosci4020028

Soni A, Henry RJ 2024. Re-calibration of flow cytometry standards for plant genome size estimation. bioRxiv: 2024.2011.2011.623134. doi: 10.1101/2024.11.11.623134

Wang L, Stegemann JP 2010. Extraction of high quality RNA from polysaccharide matrices using cetlytrimethylammonium bromide. Biomaterials 31: 1612-1618. doi: 10.1016/j.biomaterials.2009.11.024

Wang Y, et al. 2012. MCScanX: a toolkit for detection and evolutionary analysis of gene synteny and collinearity. Nucleic acids research 40: e49-e49.

Wolff J, Backofen R, Grüning B 2022. Loop detection using Hi-C data with HiCExplorer. Gigascience 11: giac061.

Wood DE, Lu J, Langmead B 2019. Improved metagenomic analysis with Kraken 2. Genome Biology 20: 257.

Zhou C, McCarthy SA, Durbin R 2023. YaHS: yet another Hi-C scaffolding tool. Bioinformatics 39: btac808. doi: 10.1093/bioinformatics/btac808
