# Supplementary material for: Haplotype-Resolved Genome of the Critically Endangered, Paleo-endemic Tree, Eidothea hardeniana
Source: Genome Biol Evol. 2026 Mar 19;18(4):evag071. doi: 10.1093/gbe/evag071 (PMC13080361; doi:10.1093/gbe/evag071)
Supplement: evag071_Supplementary_Data [file evag071_supplementary_data.zip › Supplementary_Tables_without_track_changes.docx]

**Supplementary Tables (S1-S8)**

**Haplotype-resolved genome of the critically endangered, paleo-endemic tree *Eidothea hardeniana***

Authors: Abhishek Soni^1,2^, Agnelo Furtado^1,2^, Maurizio Rossetto^3,4^, Robert J. Henry^1,5*^

^[a.soni@uq.edu.au (A.S.)](mailto:a.soni@uq.edu.au%20(A.S.)), [a.furtado@uq.edu.au](mailto:a.furdato@uq.edu.au) (A.F.),^ [^maurizio.rossetto@botanicgardens.nsw.gov.au^](mailto:maurizio.rossetto@botanicgardens.nsw.gov.au) ^(M.R.),^ [^robert.henry@uq.edu.au^](mailto:robert.henry@uq.edu.au) ^(R.J.H.)^

1. ARC Centre of Excellence for Plant Success in Nature and Agriculture, The University of Queensland, St Lucia 4072 QLD Australia
2. Centre for Crop Science, The Queensland Alliance for Agriculture and Food Science, The University of Queensland, St Lucia 4072 QLD Australia
3. Research Centre for Ecosystem Resilience, Royal Botanic Gardens, Sydney 2000 NSW Australia
4. The University of Queensland, St Lucia 4072 QLD Australia
5. VinUni Big Data Research Institute VinUniversity, Hanoi, Vietnam

*Correspondence: Prof Robert Henry (R.J.H.) [robert.henry@uq.edu.au](mailto:robert.henry@uq.edu.au)

**Table S1** Contig-level assemblies generated by hifiasm using PacBio HiFi, Hi-C, and ONT reads.

| Assembly type | Assembly length | No. of contigs | Largest contig (Mbp) | N50 (Mbp) | BUSCO (embryphyta_odb10) | T2T  contigs |
| --- | --- | --- | --- | --- | --- | --- |
| HiFi + Hi-C | 721 | 1684 | 38.6 | 23.3 | 99.4 | 0 |
| HiFi + Hi-C + ONT (unfiltered) | 671 | 879 | 36.8 | 22.8 | 99.3 | 1 |
| HiFi + Hi-C + ONT (20kb) | 698 | 835 | 59.09 | 24.04 | 99.3 | 1 |
| HiFi + Hi-C + ONT (30kb) | 696 | 804 | 59.09 | 24.08 | 99.4 | 1 |
| HiFi + Hi-C + ONT (40kb) | 682 | 691 | 59.05 | 24.19 | 99.4 | 2 |
| HiFi + Hi-C + ONT (>50kb) | 683 | 670 | 58.6 | 24.06 | 99.3 | 1 |

**Table S2** Scaffold-level assemblies generated by YaHS using PacBio HiFi, Hi-C, and ONT reads.

| Assembly type | Assembly Length (Mbp) | No. of scaffolds | Largest scaffold (Mbp) | N50 (Mbp) | BUSCO (embryphyta_odb10) | T2T  scaffolds |
| --- | --- | --- | --- | --- | --- | --- |
| HiFi + Hi-C | 721 | 1701 | 61.4 | 42 | 99.4 | 6 |
| HiFi + Hi-C + ONT (unfiltered) | 703 | 919 | 61.3 | 41.9 | 99.2 | 6 |
| HiFi + Hi-C + ONT (>20kb) | 698 | 933 | 62.08 | 44.8 | 99.3 | 5 |
| HiFi + Hi-C + ONT (>30kb) | 696 | 877 | 62 | 41.7 | 99.3 | 5 |
| HiFi + Hi-C + ONT (>40kb) | 682 | 733 | 62.4 | 45.4 | 99.4 | 5 |
| HiFi + Hi-C + ONT (>50kb) | 683 | 644 | 62.8 | 41.8 | 99.4 | 9 |

**Table S3** Repeat content profile of consensus assembly.

Total length: 614163284 bp

GC level: 39.92%

Bases masked: 331050964bp (53.90%)

| **Class/Family** | **number of elements** | | **length occupied (bp)** | | | **% of sequence** |  |  |
| --- | --- | --- | --- | --- | --- | --- | --- | --- |
|  |  |  |  |  |  |  |  |  |
| Retroelements | 109123 | | 107470748 | | | 17.5 |  |  |
| SINEs: | 487 | | 44187 | | | 0.01 |  |  |
| Penelope: | 0 | | 0 | | | 0 |  |  |
| LINEs: | 50652 | | 19139739 | | | 3.12 |  |  |
| CRE/SLACS | 0 | | 0 | | | 0 |  |  |
| L2/CR1/Rex | 6605 | | 1210294 | | | 0.2 |  |  |
| R1/LOA/Jockey | 0 | | 0 | | | 0 |  |  |
| R2/R4/NeSL | 0 | | 0 | | | 0 |  |  |
| RTE/Bov-B | 12072 | | 3908227 | | | 0.64 |  |  |
| L1/CIN4 | 31975 | | 14021218 | | | 2.28 |  |  |
| LTR elements | 57984 | | 88286822 | | | 14.38 |  |  |
| BEL/Pao | 0 | | 0 | | | 0 |  |  |
| Ty1/Copia | 19825 | | 15459018 | | | 2.52 |  |  |
| Gypsy/DIRS1 | 33095 | | 66354780 | | | 10.8 |  |  |
| Retroviral | 83 | | 14008 | | | 0 |  |  |
| DNA transposons | 16289 | | 6711320 | | | 1.09 |  |  |
| hobo-Activator | 4570 | | 2706472 | | | 0.44 |  |  |
| Tc1-IS630-Pogo | 0 | | 0 | | | 0 |  |  |
| En-Spm | 0 | | 0 | | | 0 |  |  |
| MULE-MuDR | 4702 | | 2023745 | | | 0.33 |  |  |
| PiggyBac | 0 | | 0 | | | 0 |  |  |
| Tourist/Harbinger | | 1881 | | 730997 | 0.12 | | |  |
| Other (Mirage, P-element, Transib) | | 0 | | 0 | 0 | | |  |
| Rolling-circles | | 2501 | | 1435100 | 0.23 | | |  |
| Unclassified: | | 740877 | | 203234455 | 33.09 | | |  |
| Total interspersed repeats: | |  | | 317416523 | 51.68 | | |  |
| Small RNA: | | 2083 | | 4811996 | 0.78 | | |  |
| Satellites: | | 826 | | 65899 | 0.01 | | |  |
| Simple repeats: | | 147493 | | 5918032 | 0.96 | | |  |
| Low complexity: | | 25620 | | 1419127 | 0.23 | | |  |

**Table S4:** Repeat content profile of the *Eidothea hardeniana* haplotype 1 assembly

| **Total length** | 598,174,949 bp (598,172,649 bp excl. N/X-runs) | |  |  |
| --- | --- | --- | --- | --- |
| **GC level** | 39.73% |  |  |  |
| **Bases masked** | 318,458,895 bp (53.24%) |  |  |  |
| **Category** | **Number of Elements** | **Length Occupied (bp)** | **Percentage of Sequence** |  |
| Retroelements | 114,435 | 104,021,997 | 17.39% |  |
| SINEs | 2,826 | 415,968 | 0.07% |  |
| Penelope | 0 | 0 | 0.00% |  |
| LINEs | 54,170 | 19,133,600 | 3.20% |  |
| CRE/SLACS | 0 | 0 | 0.00% |  |
| L2/CR1/Rex | 0 | 0 | 0.00% |  |
| R1/LOA/Jockey | 0 | 0 | 0.00% |  |
| R2/R4/NeSL | 0 | 0 | 0.00% |  |
| RTE/Bov-B | 13,296 | 3,839,758 | 0.64% |  |
| L1/CIN4 | 40,874 | 15,293,842 | 2.56% |  |
| LTR elements | 57,439 | 84,472,429 | 14.12% |  |
| BEL/Pao | 2,118 | 341,074 | 0.06% |  |
| Ty1/Copia | 18,274 | 15,230,859 | 2.55% |  |
| Gypsy/DIRS1 | 32,503 | 62,477,273 | 10.44% |  |
| Retroviral | 102 | 9,683 | 0.00% |  |
| DNA transposons | 13,510 | 6,155,573 | 1.03% |  |
| hobo-Activator | 4,974 | 3,184,089 | 0.53% |  |
| Tc1-IS630-Pogo | 0 | 0 | 0.00% |  |
| En-Spm | 0 | 0 | 0.00% |  |
| MULE-MuDR | 2,536 | 1,207,683 | 0.20% |  |
| PiggyBac | 0 | 0 | 0.00% |  |
| Tourist/Harbinger | 2,495 | 698,046 | 0.12% |  |
| Other (Mirage, P-element, Transib) | 0 | 0 | 0.00% |  |
| Rolling-circles | 5,711 | 1,916,706 | 0.32% |  |
| Unclassified | 728,341 | 197,887,024 | 33.08% |  |
| Total interspersed repeats | – | 308,064,594 | 51.50% |  |
| Small RNA | 2,374 | 1,498,882 | 0.25% |  |
| Satellites | 0 | 0 | 0.00% |  |
| Simple repeats | 146,461 | 5,819,145 | 0.97% |  |
| Low complexity | 24,805 | 1,367,541 | 0.23% |  |

| **Table S5:** Repeat content profile of the *Eidothea hardeniana* haplotype 2 assembly | | | | |
| --- | --- | --- | --- | --- |
| total length: | 595787266 bp | |  |  |
| GC level | 39.73% |  |  |  |
| bases masked: | 318422327 bp (53.45%) | | |  |
| **Class / Family** | **Number of Elements** | **Length Occupied (bp)** | **% of Sequence** |  |
| Retroelements | 124,609 | 110,010,579 | 18.46% |  |
| SINEs | 1,261 | 678,810 | 0.11% |  |
| Penelope | 0 | 0 | 0.00% |  |
| LINEs | 42,754 | 18,648,733 | 3.13% |  |
| CRE/SLACS | 0 | 0 | 0.00% |  |
| L2/CR1/Rex | 0 | 0 | 0.00% |  |
| R1/LOA/Jockey | 0 | 0 | 0.00% |  |
| R2/R4/NeSL | 0 | 0 | 0.00% |  |
| RTE/Bov-B | 9,643 | 3,592,215 | 0.60% |  |
| L1/CIN4 | 32,804 | 14,976,499 | 2.51% |  |
| LTR elements | 80,594 | 90,683,036 | 15.22% |  |
| BEL/Pao | 0 | 0 | 0.00% |  |
| Ty1/Copia | 45,404 | 22,991,228 | 3.86% |  |
| Gypsy/DIRS1 | 31,114 | 61,426,491 | 10.31% |  |
| Retroviral | 1,205 | 927,925 | 0.16% |  |
| DNA transposons | 15,338 | 6,884,960 | 1.16% |  |
| hobo-Activator | 6,034 | 3,330,301 | 0.56% |  |
| Tc1-IS630-Pogo | 0 | 0 | 0.00% |  |
| En-Spm | 0 | 0 | 0.00% |  |
| MULE-MuDR | 3,596 | 1,807,056 | 0.30% |  |
| PiggyBac | 0 | 0 | 0.00% |  |
| Tourist/Harbinger | 1,710 | 741,061 | 0.12% |  |
| Other (Mirage, P-element, Transib) | 0 | 0 | 0.00% |  |
| Rolling-circles | 2,505 | 1,384,403 | 0.23% |  |
| Unclassified | 720,170 | 190,386,283 | 31.96% |  |
| Total interspersed repeats | - | 307,281,822 | 51.58% |  |
| Small RNA | 3,505 | 2,821,128 | 0.47% |  |
| Satellites | 0 | 0 | 0.00% |  |
| Simple repeats | 145,327 | 5,878,791 | 0.99% |  |
| Low complexity | 23,317 | 1,320,831 | 0.22% |  |
|  |  |  |  |  |
|  |  |  |  |  |

**Table S6:** Structural annotation results for consensus genome, haplotype 1 genome and haplotype 2 genome generated with BRAKER

| **Metric** | **consensus** | **hap1** | **hap2** |
| --- | --- | --- | --- |
| Number of gene | 26677 | 26315 | 26078 |
| Number of mrna | 30411 | 29712 | 29416 |
| Number of cds | 30411 | 29712 | 29416 |
| Number of exon | 165393 | 160739 | 159849 |
| Number of intron | 134982 | 131027 | 130433 |
| Number of start_codon | 30407 | 29708 | 29410 |
| Number of stop_codon | 30410 | 29711 | 29414 |
| Number of exon in cds | 165393 | 160739 | 159849 |
| Number of intron in cds | 134982 | 131027 | 130433 |
| Number of intron in exon | 134982 | 131027 | 130433 |
| Number of intron in intron | 112036 | 108690 | 108192 |
| Number gene overlapping | 79 | 63 | 73 |
| Number of single exon gene | 6849 | 6766 | 6607 |
| Number of single exon mrna | 7465 | 7375 | 7175 |
| mean mrnas per gene | 1.1 | 1.1 | 1.1 |
| mean cdss per mrna | 1 | 1 | 1 |
| mean exons per mrna | 5.4 | 5.4 | 5.4 |
| mean introns per mrna | 4.4 | 4.4 | 4.4 |
| mean start_codons per mrna | 1 | 1 | 1 |
| mean stop_codons per mrna | 1 | 1 | 1 |
| mean exons per cds | 5.4 | 5.4 | 5.4 |
| mean introns in cdss per mrna | 4.4 | 4.4 | 4.4 |
| mean introns in exons per mrna | 4.4 | 4.4 | 4.4 |
| mean introns in introns per mrna | 3.7 | 3.7 | 3.7 |
| Total gene length | 152453883 | 151196725 | 150567647 |
| Total mrna length | 184580639 | 179823427 | 178853367 |
| Total cds length | 40479703 | 39298114 | 38953929 |
| Total exon length | 40479703 | 39298114 | 38953929 |
| Total intron length | 144100936 | 140525313 | 139899438 |
| Total start_codon length | 91206 | 89109 | 88214 |
| Total stop_codon length | 91205 | 89106 | 88217 |
| Total intron length per cds | 144100936 | 140525313 | 139899438 |
| Total intron length per exon | 144100936 | 140525313 | 139899438 |
| Total intron length per intron | 18558626 | 17963953 | 17878045 |
| mean gene length | 5714 | 5745 | 5773 |
| mean mrna length | 6069 | 6052 | 6080 |
| mean cds length | 1331 | 1322 | 1324 |
| mean exon length | 244 | 244 | 243 |
| mean intron length | 1067 | 1072 | 1072 |
| mean start_codon length | 2 | 2 | 2 |
| mean stop_codon length | 2 | 2 | 2 |
| mean cds piece length | 244 | 244 | 243 |
| mean intron in cds length | 1067 | 1072 | 1072 |
| mean intron in exon length | 1067 | 1072 | 1072 |
| mean intron in intron length | 165 | 165 | 165 |
| Longest gene | 307639 | 307639 | 366385 |
| Longest mrna | 307639 | 307639 | 307639 |
| Longest cds | 16461 | 16461 | 16461 |
| Longest exon | 8034 | 8034 | 8034 |
| Longest intron | 307091 | 307091 | 307091 |
| Longest start_codon | 3 | 3 | 3 |
| Longest stop_codon | 3 | 3 | 3 |
| Longest cds piece | 8034 | 8034 | 8034 |
| Longest intron into cds part | 307091 | 307091 | 307091 |
| Longest intron into exon part | 307091 | 307091 | 307091 |
| Longest intron into intron part | 8034 | 8034 | 8034 |
| Shortest gene | 105 | 112 | 33 |
| Shortest mrna | 54 | 54 | 33 |
| Shortest cds | 9 | 9 | 9 |
| Shortest exon | 1 | 1 | 1 |
| Shortest intron | 22 | 24 | 35 |
| Shortest start_codon | 1 | 1 | 1 |
| Shortest stop_codon | 1 | 1 | 1 |
| Shortest cds piece | 1 | 1 | 1 |
| Shortest intron into cds part | 22 | 24 | 35 |
| Shortest intron into exon part | 22 | 24 | 35 |
| Shortest intron into intron part | 5 | 5 | 5 |

**TableS7:** BUSCO assessment of gene model completeness for the consensus and haplotype assemblies of *Eidothea hardeniana* against database embryophyta_odb10

|  |  | consensus | hap1 | hap2 |
| --- | --- | --- | --- | --- |
| Complete BUSCOs (C) | | 97.7% | 97.2% | 96.8% |
| Complete and single copy | | 87.4% | 86.9% | 10.4% |
| Complete and duplicated | | 10.3% | 10.3% | 10.4% |
| Fragmented BUSCOs (F) | | 0.4% | 0.4% | 0.6% |
| Missing BUSCOs (M) | | 1.9% | 2.4% | 2.6% |
| Total BUSCO groups | | 1614 | 1614 | 1614 |

**Table S8:** Functional annotation of predicted genes in the consensus and haplotype assemblies of *Eidothea hardeniana*

|  | consensus | hap1 | hap2 |
| --- | --- | --- | --- |
| Total coding sequences | 30120 | 29712 | 29416 |
| Coding sequences with GO annotation | 24649 | 24304 | 24011 |
| Coding sequences with GO mapping only | 163 | 163 | 167 |
| Coding sequences with Blast hit only | 4760 | 4726 | 4699 |
| Coding sequences with no Blast hits | 548 | 519 | 539 |
| No Blast hit sequences with coding potential | 479 | 457 | 479 |
